# A molecular basis for water motion detection by the mechanosensory lateral line of zebrafish

**DOI:** 10.1038/s41467-017-01604-2

**Published:** 2017-12-21

**Authors:** Shih-Wei Chou, Zongwei Chen, Shaoyuan Zhu, Robin W. Davis, Jiaqi Hu, Li Liu, Carol A. Fernando, Kayla Kindig, William C. Brown, Ruben Stepanyan, Brian M. McDermott

**Affiliations:** 1 Department of Otolaryngology–Head and Neck Surgery, Case Western Reserve University School of Medicine, Cleveland, OH 44106 USA; 2 Department of Biology, Case Western Reserve University, Cleveland, OH 44106 USA; 3 Department of Neurosciences, Case Western Reserve University School of Medicine, Cleveland, OH 44106 USA; 4 Department of Genetics and Genome Sciences, Case Western Reserve University School of Medicine, Cleveland, OH 44106 USA

## Abstract

Detection of water motion by the lateral line relies on mechanotransduction complexes at stereocilia tips. This sensory system is comprised of neuromasts, patches of hair cells with stereociliary bundles arranged with morphological mirror symmetry that are mechanically responsive to two opposing directions. Here, we find that transmembrane channel-like 2b (Tmc2b) is differentially required for mechanotransduction in the zebrafish lateral line. Despite similarities in neuromast hair cell morphology, three classes of these cells can be distinguished by their Tmc2b reliance. We map mechanosensitivity along the lateral line using imaging and electrophysiology to determine that a hair cell’s Tmc2b dependence is governed by neuromast topological position and hair bundle orientation. Overall, water flow is detected by molecular machinery that can vary between hair cells of different neuromasts. Moreover, hair cells within the same neuromast can break morphologic symmetry of the sensory organ at the stereocilia tips.

## Abstract (web summary)

In fish, water motion is detected by mechanosensitive hair cells located in the lateral line. Here the authors show that the molecular machinery for mechanotransduction, including transmembrane channel-like 2b (Tmc2b), varies depending on both hair cell location and hair bundle orientation.

## Introduction

Perception of water motion in fish is initiated by the deflection of mechanosensitive hair bundles that are topographically fixed on the animal’s surface in the lateral line system^1–3^. For this sense, mechanotransduction occurs at the tips of cellular protrusions, the stereocilia, which have increasing heights and are interconnected by tip links^4^ to comprise the hair cell’s bundle. Mechanically gated ion channels at the tips of the shorter stereocilia are opened when a bundle is deflected in the direction of the tallest stereocilia, permitting cation entry^5,6^.

The lateral line system detects two types of water movement through receptive neuromasts on the animal’s surface: water velocity^7,8^ and minuscule water vibrations^7,9^. Though the anterior lateral line (ALL) bespeckles the larval head in a complex pattern, the posterior lateral line (PLL) generally runs evenly along the trunk and tail^10^. Each neuromast houses a sheet of hair cells organized with planar cell polarity (PCP) in a mirror symmetrical arrangement of hair bundles^2^, half in each direction. Hair cells with similarly facing hair bundles are innervated by a dedicated afferent neuron, which carries that group’s information centrally^11,12^. Each PLL neuromast’s axis of mirror symmetry is oriented either parallel or perpendicular to the anterior–posterior (A–P) body axis^13^. Understanding the molecular basis of water motion detection requires identification of molecules at the tips of stereocilia that are necessary for mechanotransduction and relating them to the intricate topology of the lateral line. An intriguing class of proteins that may play a role in this relationship are the members of the transmembrane channel-like (Tmc) protein family, known for their intimate association with the mechanotransduction process^14–21^.

## Results and Discussion

### A lateral line with two general axes of best sensitivity

**Fig. 1:**
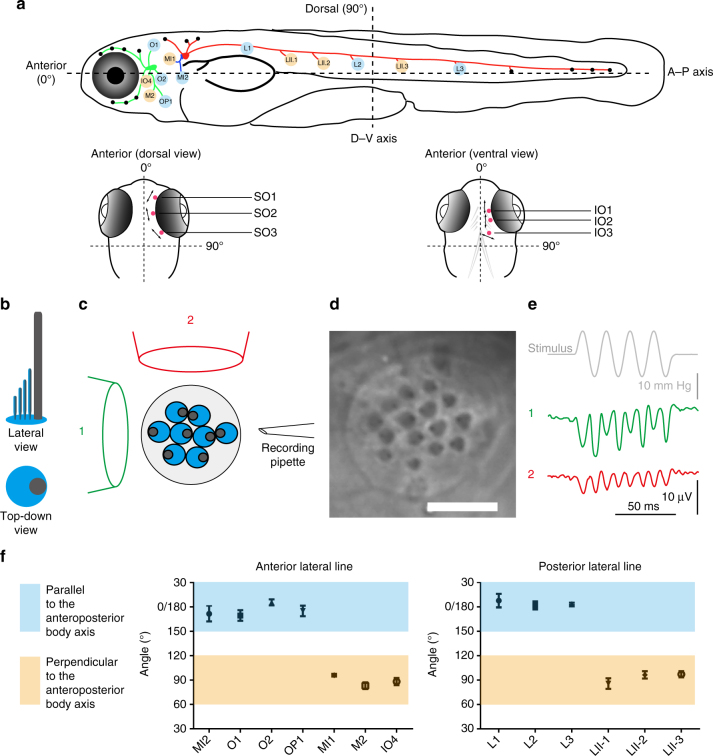
Axes of best sensitivity for anterior and posterior lateral line neuromasts. **a** Schematic of the zebrafish lateral line at ~6 dpf. (top) Side view. Neuromasts that have been scored are blue or tan and correspond to the graph below. ALL(green), PLL(red), and medial–lateral-line (blue) ganglia contact neuromasts. (bottom) Dorsal and ventral views of the head. Supraorbital (SO) neuromasts 1 to 3 and infraorbital (IO) neuromasts 1 to 3 are marked in red. The mean angle of the orientation of the axis of best sensitivity relative to the A–P body axis of each neuromast is presented as a double-headed arrow. **b**–**e** Evaluation of the axis of best sensitivity for a neuromast. **b** Schematics of hair bundles. Gray and blue protrusions are a kinocilium and stereocilia, respectively. **c** Experimental configuration: a top-down view of a neuromast with a fluid jet pipette that provides stimuli (1) parallel to the axis  along which the bevel-shaped hair bundles face opposite directions or (2) orthogonal to that axis. The pipettes in the diagram are not to scale. **d** DIC image of hair bundles of a posterior neuromast from which stimulus-evoked microphonic potentials were recorded. Scale bar = 5 μm. **e** Recordings from an A–P oriented L3 neuromast with fluid jet pipettes stimulating, serially, from two different directions depicted in **c**. **f** Graphs of mean angle of the orientation of the axis of best sensitivity relative to the A–P body axis of each anterior and posterior neuromast position ± SEM (*n* *= *4–7). Each axis with a range from 60° to 120° from the A–P body axis is defined perpendicular (tan). Each axis with a range from 0° to 30° or 150° to 180° from the A–P body axis is defined as parallel (blue). ALL nomenclature: opercular (OP), mandibular (M), infraorbital (IO), supraorbital (SO), middle (MI) and otic (O). PLL nomenclature: L neuromasts are A–P oriented; LII neuromasts are D–V oriented. These different neuromast types are derived from different migrating primordia during development

Establishing the molecular basis of lateral line function first requires clarifying the anatomical relationships that may exist between neuromasts of the zebrafish head and those of the trunk and tail. Most neuromasts of the head, trunk, and tail lie on approximate single planes or sheets on either lateral side of the fish (Fig. 1a, top); these were the first to be characterized, as they are the most abundant. The topologically complex larval ALL may have neuromasts with organizations similar to cranial neuromasts of other adult fish species, with axes of best sensitivity oriented to multiple directions^22^. Alternatively, this lateral line may have its neuromasts oriented along just two  axes.

Addressing this question required multiple steps. First, we functionally tested whether the axis of best sensitivity within a single neuromast was the axis in which the bevel-shaped hair bundles face opposite directions. Specifically, we asked if stimuli provided along this axis had similar or attenuated responses to those of stimuli administered from an orthogonal direction (Fig. 1b, c). For this experiment, we evaluated the quality of mechanotransduction in neuromast organs by measuring microphonic potentials evoked by sinusoidal stimuli^23^. In normal neuromasts of wild-type larvae, a 2f (frequency) response is generally observed because each phase of stimulation activates approximately half of the hair cells, whereas the rest are inhibited. This telltale signature results from the presence of two groups of hair cells with hair bundles facing opposite directions responding to opposing stimuli^2,24^ (Fig. 1b, c). The responses from stimuli that we provided along the axis with opposite facing hair bundles were larger than responses from stimuli perpendicular to this axis (Fig. 1d, e), indicating that the axis in which the hair bundles are opposing each other is the axis of best sensitivity for each larval neuromast.

Next, by labeling hair bundle actin of larvae with phalloidin, we showed that most anterior neuromasts’ axes of mirror symmetry are parallel or perpendicular to the A–P axis, similar to the PLL (Fig. 1a, top, f; Supplementary Fig. 1). Using the same method, we determined the axes of best sensitivity for a minor subset of neuromasts that are positioned superior or inferior to the eye (Fig. 1a, bottom). Interestingly, the axes of best sensitivity for the neuromasts above the eye were tangential to the perimeter of the eye (Fig. 1a, bottom left). For three neuromasts below the eye, the axes of best sensitivity were in A–P orientations for two, and one had an axis that was positioned obliquely to the A–P axis (Fig. 1a, bottom right).

Taken together, these data suggest that the majority of neuromasts have axes of best sensitivity that set up a Cartesian coordinate system to organize water motion information along the whole body. Moreover, the consolidation of many potential neuromast sensitivities to just two axes may allow for economical use of a limited number of larval neuromasts, which could facilitate an additive response from a population of neuromasts with hair cells that face the same direction. However, this anatomical arrangement creates a potential conundrum. A water flow stimulus from the front, caused by normal swimming, may require a different response than one originating from the rear, possibly caused by a predator. To detect this difference, neuromasts sensitive in the A–P plane may require breaking morphologic symmetry to differentially encode directional information. We reasoned that a direct mechanism to modify neuromast responses to flow from different directions would be to alter the mechanotransduction apparatus itself.

### Posterior neuromasts require Tmc2b for mechanotransduction

**Fig. 2:**
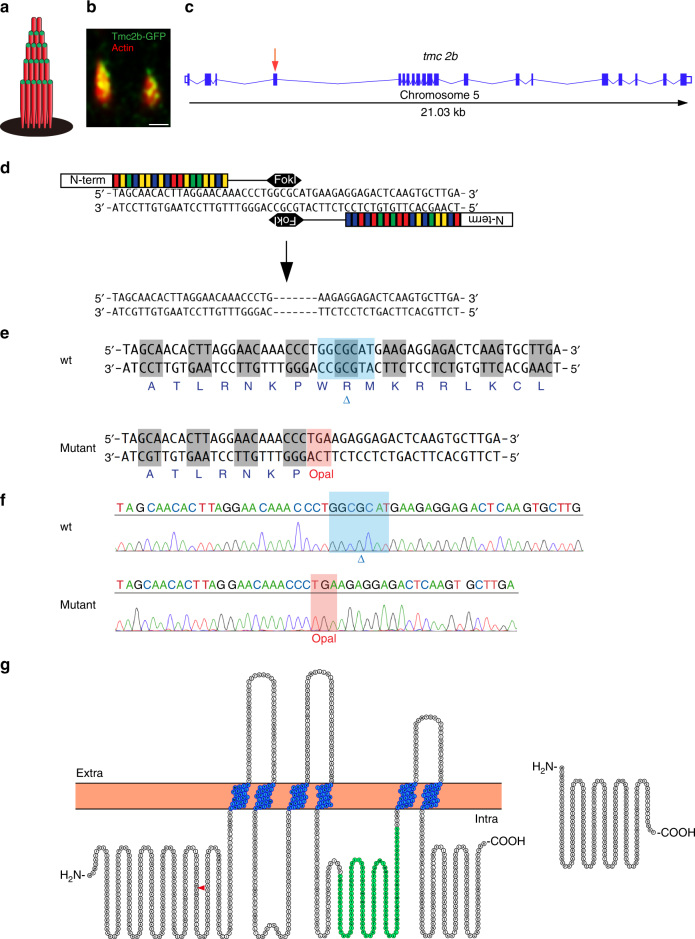
Localization of Tmc2b and TALEN-mediated disruption of the *tmc2b* gene. **a** Schematic of the hair bundle. **b** Tmc2b localizes to the tips of stereocilia in a neuromast. Two lateral line hair cells expressing Tmc2b-GFP (green) demonstrate that the fusion protein localizes to the tips of β-actin-mCherry-labeled stereocilia (red). Scale bar = 0.5 μm. This pattern was observed in 130 of 381 hair cells of somatic transgenics. In transgenic hair cells with weak expression, localization to stereocilia was difficult to recognize. **c** Graphical representation of the *tmc2b* genomic locus in zebrafish. Putative exons and splice sites are displayed. Red arrow marks the targeted exon, exon 4. **d** Segment of exon 4 subjected to genome editing. Two differently engineered TALENs bind their corresponding half-sites to enable FokI dimerization and DNA cleavage. Mutagenesis deleted seven nucleotides. **e** Amino acid sequences of wild-type and mutant proteins. The alteration results in an opal mutation upstream of all putative transmembrane domains. **f** Sequencing results of mutagenized and control loci. Blue highlight and blue delta indicates deleted 7-nucleotide stretch absent in mutant. Red highlight denotes the opal mutation that was generated at the site of the TALEN targeting. **g** (left) Topographical representation of the Tmc2b protein. Red arrowhead indicates point of introduced mutation. Amino acids of putative transmembrane domains are labeled in blue and the TMC domain is in green. (right) Predicted truncated product of mutation

Tmc proteins are hypothesized elements of the mechanotransduction apparatus of the lateral line based on established roles of orthologous proteins in mammalian hearing^14,16–21^ and expression patterns and dominant negative studies in fish^15^. According to RNA in situ hybridization studies of zebrafish larva, *tmc2b* is robustly expressed in hair cells of the lateral line, but the mRNAs of the paralogs *tmc2a* and *tmc1* are present at much lower levels and cannot be detected by this method. However, their presence is nonetheless observed by more sensitive molecular biological techniques^15^. Because of the higher level of expression and the known function of mouse  orthologous proteins, we explicitly tested the notion that Tmc2b is necessary for mechanotransduction in the lateral line. First, by transgenesis, we showed that full-length Tmc2b fused to GFP localizes to the tips of neuromast stereocilia (Fig. 2a, b), fulfilling a key requirement of being a component of the mechanotransduction apparatus in zebrafish hair cells. Second, to test whether Tmc2b is necessary for hair cell mechanotransduction in the lateral line system, we performed genome editing with transcription activator-like effector nucleases (TALENs), synthetic restriction enzymes generated by fusing a TAL effector DNA-binding domain to a DNA-cleavage domain (Fig. 2c, d)^25^. Targeting with the TAL effector pair resulted in a mutant with a 7-bp deletion in exon 4, which was bred to homozygosity (Fig. 2e, f). The *tmc2b* mutation is predicted to result in a frame shift that yields a premature opal stop codon (UGA), creating a truncated protein of 148 aa, as opposed to the wild-type protein of 940 aa. The mutation is expected to stop all putative transmembrane domains from being produced to create a null (Fig. 2e, g).

**Fig. 3:**
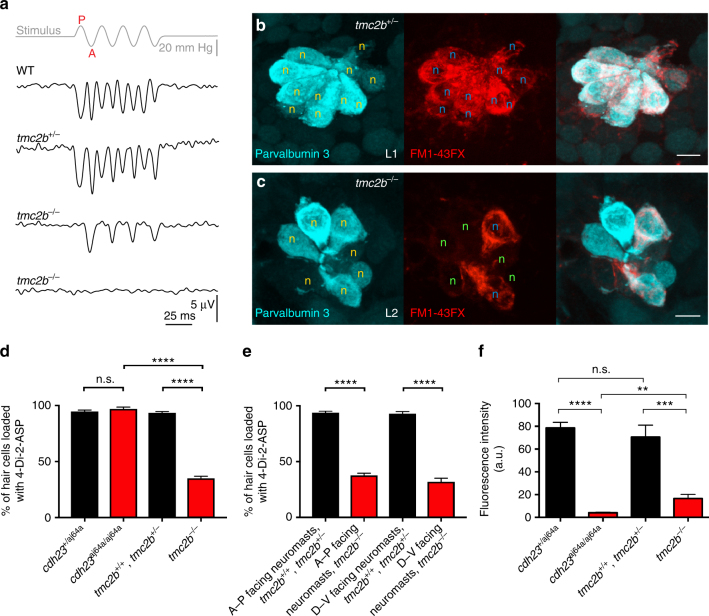
Variable dependence on Tmc2b for mechanotransduction in posterior neuromast hair cells. **a** Extracellular recordings of microphonic potentials measured from posterior neuromasts with A-P orientations of 6-dpf zebrafish larvae are displayed. The responses in *tmc2b* ^*−/−*^ are often absent, bottom trace (*n* = 9/15); however, several responses (*n* = 6/15) are highly asymmetric, with weakened amplitude for one direction of stimulus and no response for the other direction, penultimate trace. Deflection anteriorly (A) or posteriorly (P). **b**, **c** Qualitative confocal images of hair cells labeled with FM1-43FX (red), to assess mechanotransduction function, and parvalbumin 3 (cyan)^50^, as a counter label, to visualize mature hair cells. **b** A posterior neuromast from a *tmc2b* ^*+/−*^ animal demonstrates co-labeling of all hair cells. **c** In a *tmc2b* ^*−/−*^ animal, a posterior neuromast contains hair cells that label with FM1-43FX and a subset that do not (label:no label; 2:5). Orange n, hair cells labeled with parvalbumin 3 antiserum. Blue n, hair cells that load with FM1-43FX. Green n, hair cells that do not load. Scale bar = 5 μm. **d**, **e** Percentages of hair cells within PLL neuromasts that label with 4-Di-2-ASP. **d** In neuromasts of *tmc2b* ^*+/+*^ and *tmc2b* ^*+/−*^ animals, most hair cells take up fluorophore. Approximately 35% of hair cells in each PLL neuromast of *tmc2b* ^*−/−*^ fish take up 4-Di-2-ASP. In contrast, in *cdh23* ^*aj64a/aj64a*^ mutants, all hair cells take up dye, though at much lower quantities than wild-type animals (see **f** and Supplementary Fig. 4b). **** Kruskal–Wallis test *P* value < 0.0001. *n* values ≥ 20. **e** In mutants, A–P-facing and D–V-facing posterior neuromasts have similar percentages of hair cells that gather fluorophore, ~38 and 32%, respectively. ****Student’s *t*-test *P* value < 0.0001. *n* values ≥ 23. **f** Mean fluorescence intensity of 4-Di-2-ASP uptake of the brightest cell per neuromast. *n* values ≥ 17. Kruskal–Wallis test ****P < 0.0001, ***P = 0.001, **P = 0.0215

Initially, we appraised the quality of mechanotransduction in neuromast organs by measuring stimulus-evoked microphonic potentials^23^. In posterior neuromasts with A–P orientations in larvae at 6-days postfertilization (dpf), delivery of a sinusoidal stimulus evoked either no extracellular receptor potentials in *tmc2b* ^*−/−*^ larvae (9 of 15) (Fig. 3a, bottom trace), or a greatly attenuated response (6 of 15) (Fig. 3a, third trace), relative to controls (Fig. 3a first and second trace) (mean potential of *tmc2b* ^*−/−*^ responders ± SEM = 5.2 ± 1.1 µV (*n* = 6), mean potential of *tmc2b* ^+/*−*^ = 8.4 ± 0.34 µV (*n* = 15), and mean potential of *tmc2b* ^+/+^ (WT) = 8.0 ± 0.81 µV (*n* = 5)). Interestingly, the attenuated responses were often asymmetrical, with the 2f response replaced with a 1f response (Fig. 3a, third trace), which was stronger when the hair bundles were deflected anteriorly. In mutants, no defects in hair bundle morphology (Supplementary Fig. 2a–d) or hearing (Supplementary Fig. 2f, g) were observed. However, posterior neuromasts of mutants had a mild reduction in hair cell numbers (approximately one-third decrease, 6 dpf; Supplementary Fig. 2c–e; Supplementary Fig. 3 **)**. These studies demonstrate that Tmc2b is necessary for normal mechanotransduction in posterior neuromast organs with A–P orientations.

### Posterior neuromast hair cells differentially require Tmc2b

To test whether mutation of *tmc2b* reduces mechanotransduction in some hair cells more than others in posterior neuromasts with A–P orientations, we visualized the uptake of cationic styryl dye FM1-43FX. This fluorescent molecule transits through the hair cell’s mechanotransduction channel, serving as a qualitative indicator of channel function when fixed^26^ (Fig. 3b, c). *Tmc2b* ^*−/−*^ mutants at 6-dpf showed exceptionally uneven FM1-43FX loading between cells in the same neuromast (Fig. 3c), indicating that the molecular components that are directly or indirectly involved in mechanotransduction can differ between hair cells.

We next examined whether residual mechanotransduction channel function was uniform or heterogeneous among permissive hair cells in posterior neuromasts with A–P orientations in mutants using a multistep process. Quantitative 4-(4-diethylaminostyryl)-1-methylpyridinium iodide (4-Di-2-ASP) hair cell uptake assays were used because this fluorophore traverses mechanotransduction channels^27,28^. First, we determined the percentages of hair cells that loaded 4-Di-2-ASP in posterior neuromasts in 6-dpf larvae. 94 ± 1.2% (*n* = 55) of control hair cells loaded fluorophore; in contrast, 35 ± 1.8% (*n* = 48) of *tmc2b* ^*−/−*^ hair cells loaded (Fig. 3d), confirming Tmc2b is absolutely required for a subpopulation of hair cells within a single neuromast. For comparison, we determined the percentage of hair cells that took up fluorophore in posterior neuromasts from *cdh23* ^*aj64a/aj64a*^ mutants, which lack functional tip links^29^, and found that 97 ± 1.6% (*n* = 27) of hair cells took up 4-Di-2-ASP, but at a much reduced level (Fig. 3d, f; Supplementary Fig. 4b). Interestingly, this indicates mechanotransduction is uniformly dependent on Cdh23 but heterogeneously dependent on Tmc2b. We also asked whether posterior neuromasts with D–V orientations had similar or different percentages of hair cells with Tmc2b reliance as A–P-oriented neuromasts, and found that both classes of neuromasts were similarly impacted (Fig. 3e).

Second, the quality of mechanotransduction was roughly ascertained by determining the relative amount of 4-Di-2-ASP that could enter hair cells by measuring the fluorescence intensity of the brightest cell for each neuromast through live imaging. In *tmc2b* ^*−/−*^ animals, the cells eligible to take up the dye did so at a much reduced efficiency, ~fourfold less than controls, when the fluorescence signals were compared (Fig. 3f). This indicates a class of hair cell that is partially dependent on Tmc2b. The decreased dye uptake correlated with reduced microphonic potentials in *tmc2b* ^*−/−*^ mutants; all *tmc2b* ^*−/−*^ mutants (15/15) examined by both assays demonstrated reduced dye uptake and microphonic potentials, but all controls (10/10) had normal uptake and microphonic potentials.

Third, with high resolution, we examined whether mechanotransduction properties are uniform in functional hair cells or if there is a range of mechanotransduction function in neuromasts of *tmc2b* ^*−/−*^ animals. 4-Di-2-ASP presence was evaluated in all of the individual hair cells within a neuromast organ using quantitative confocal microscopy (Supplementary Fig. 4a). In *tmc2b* ^*+/−*^ fish, every L1 hair cell loaded the fluorophore, displaying a range of intensities that were all above background. In contrast, in a *tmc2b* ^*−/−*^ neuromast, most hair cells’ intensities were not above background, and the two cells that did transduce did so at a reduced level (Supplementary Fig. 4a). To inspect each neuromast for hair cells that took up the fluorophore, even at minuscule levels, we increased the optical power of the imaging by increasing the gain. For the same neuromast imaged in Supplementary Fig. 4a, we observed a several-fold increase in fluorescence levels of the two cells that did transduce (Supplementary Fig. 4b). Overall, these findings demonstrate that there is a subpopulation of hair cells that are entirely dependent on a single Tmc family member, which strikingly contrasts with the requirement in the mammalian ear, where there is redundancy between TMC1 and TMC2^16,18,19^. Moreover, within the population of hair cells that still transduce, there is variability in transduction, yet all show some Tmc2b reliance, suggesting potential heterogeneity in the mechanotransduction channel, or its ancillary proteins, between hair cells within a single neuromast.

To determine if there was upregulation of *tmc2b* paralogs *tmc1* or *tmc2a*, we assessed relative expression levels of cognate mRNAs in wild-type and *tmc2b* ^*−/−*^ mutant zebrafish. For each of these genes, expression in the mutants decreased slightly relative to controls as determined by RT-PCR experiments (Supplementary Fig. 5). This is an expected result that correlates with the decrease in hair cell numbers in the mutants because fewer hair cells would naturally lead to fewer total transcripts. These findings indicate that these genes are not upregulated in response to genetic inactivation of *tmc2b*.

### Spatial position controls head neuromast mechanotransduction

**Fig. 4:**
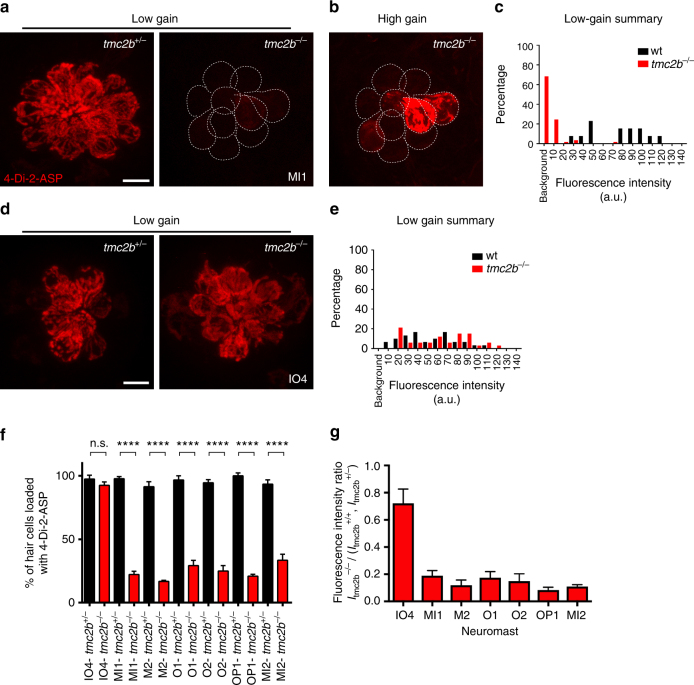
Spatial positioning of anterior neuromasts regulates diverse Tmc2b dependence. **a** Confocal micrographs of MI1 hair cells incubated with 4-Di-2-ASP from *tmc2b* ^*+/−*^ (left) or *tmc2b* ^*−/−*^ (right) animals at 6 dpf, viewed under low gain. **b** Hair cells from **a**, right, viewed under high gain. **c** Percentages of MI1 hair cells at different fluorescence intensities (*n* = 5). **d** Images and **e** percentages of hair cells at different fluorescence intensities from IO4 neuromasts (*n* = 5). **f** Percentages of hair cells of ALL neuromasts that take up 4-Di-2-ASP. ****One-way ANOVA with Holm-Sidak’s multiple comparisons test *P* *< *0.0001. *n* values (het/homo): IO4 = 6/4, MI1 = 7/5, M2 = 5/3, O1 = 5/2, O2 = 5/4, OP1 = 5/3, MI2 = 5/5. **g** Mean whole-neuromasts normalized fluorescence intensity ratios, *I*_*tmc2b*_^*−/−*^/ (*I*_*tmc2b*_^+/+^, *I*_*tmc2b*_^+/*−*^). *n* values ≥ 5. Scale bar = 6 µm

Because the geometry of neuromast position is distinct between neuromasts of the head and those of the trunk and tail, complex vs. linear (Fig. 1a, top)^10,30^, we examined whether patterns of mechanotransduction function varied in the ALL of *tmc2b* ^*−/−*^ animals at 6 dpf using 4-Di-2-ASP. MI1 neuromasts demonstrated a similar pattern of partial dependence as posterior neuromasts, with most hair cells absolutely requiring Tmc2b, and a smaller subset, 22%, still able to load fluorophore (Fig. 4a–c,f). These cells took up fluorophore at a reduced efficiency, ~fivefold less than controls (Fig. 4g). Similar patterns of fluorophore entry were observed for most neuromasts of the head (Fig. 4f, g). In sharp contrast, neuromast IO4 was minimally impacted by the *tmc2b* mutation (Fig. 4d–g). The number of hair cells that took up 4-Di-2-ASP was similar to control values and the fluorescence intensity was mildly reduced (normalized mean fluorescence intensity ratio of whole neuromasts from *tmc2b* ^*−/−*^ larvae is 0.72 ± 0.1 (*n* = 15)), indicating that hair cells of this neuromast are minimally reliant on, or independent of, Tmc2b for mechanotransduction (also see microphonic potential values for IO4 below). Additionally, the number of IO4 hair cells are the same in *tmc2b* ^*−/−*^ mutants and wild-type animals, showing that this gene is not necessary to sustain this assemblage of hair cells (Supplementary Fig. 3c).

### Hair bundle orientation influences mechanotransduction

**Fig. 5:**
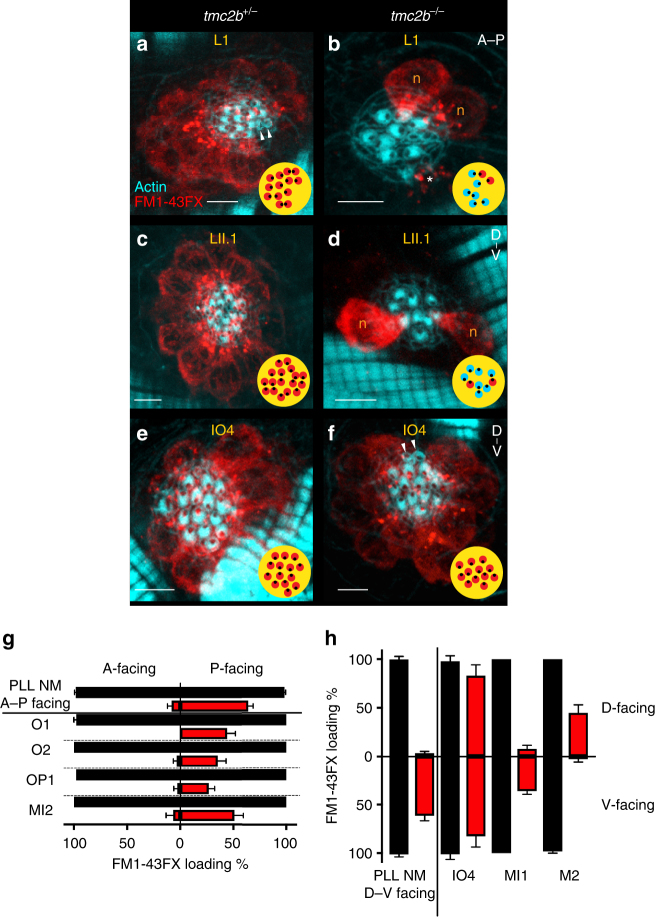
Hair cell PCP and neuromast position govern dependence of mechanotransduction channel function on Tmc2b. **a**–**f** Confocal images of hair cells from L1, LII.1, and IO4 neuromasts of *tmc2b* ^*+/−*^ or *tmc2b* ^*−/−*^ animals. FM1-43FX (red) uptake reveals functional channels, and phalloidin (cyan) shows hair bundle polarity. Qualitative maps (yellow) of micrographs from *tmc2b* ^*−/−*^ mutants show that subpopulations of posterior- and ventral-facing hair bundles preferentially function (red) in L1 and LII.1, respectively; in contrast, hair cell uptake in IO4 is non-biased. **g** In PLL neuromasts with A–P orientations of *tmc2b* ^*−/−*^ mutants, the vast majority (*n* = 21) of FM1-43FX uptake is by hair bundles that are posterior facing (P-facing). Similarly, in the A–P-oriented neuromasts of the ALL of *tmc2b* ^*−/−*^ (O1, O2, OP1, and MI2), the overwhelming majority of FM1-43FX uptake is by P-facing hair cells (*n* ≥ 5, except for O1, *n* = 4). Red bars represent *tmc2b* ^*−/−*^, and black bars signify *tmc2b* ^*+/+*^ and *tmc2b* ^*+/−*^. **h** In the PLL neuromasts with D–V orientations of *tmc2b* ^*−/−*^ mutants, ventral-facing (V-facing) hair cells dominate the population that uptakes the fluorophore (*n* = 16). In ALL neuromasts with D–V orientations of *tmc2b* ^*−/−*^, the patterns of hair cells that take up FM1-43FX are complex. For IO4, there is no significant difference between the numbers of D- and V-facing hair cells that loaded with FM1-43FX. The FM1-43FX loading percentages are 82.6 ± 11.1 % (*n* = 5) for V-facing hair cells and 83.0 ± 11.1 % (*n* = 5) for D-facing hair cells in *tmc2b* ^*−/−*^, respectively. One-way ANOVA with Holm-Sidak’s multiple comparisons test, *P* *= *0.9986. D–V-oriented neuromasts MI1 and M2 have opposite PCP-related loading preferences in mutants. In MI1 and M2 of *tmc2b* ^*−/−*^ animals, the percentages of V-facing hair cells that load with FM1-43FX are 36.1 ± 3.4% (*n* = 6) and 2.9 ± 2.9% (*n* = 5), respectively. Whereas, the percentages of D-facing hair cells from MI1 and M2 that load with FM1-43FX are 7.9 ± 3.6% (*n* = 6) and 44.9 ± 8.3% (*n* = 5), respectively (see Supplementary Table 1 for statistics). Corresponding images of MI1 and M2 displayed in Supplementary Fig. 7. Scale bar = 5 μm. Arrowheads, immature hair cells that do not take up FM1-43FX. *dying hair cell

To further explore the molecular and cellular logic of heterogeneity in mechanotransduction of the PLL, we co-labeled *tmc2b* ^+/*−*^ and *tmc2b* ^*−/−*^ larvae with fluorophore-coupled phalloidin and FM1-43FX to reveal the potential relationship between hair bundle orientation and residual mechanotransduction channel function. In controls, hair cells of posterior neuromasts L1 and LII.1, with A–P- and D–V-facing hair bundles, respectively, took up FM1-43FX similarly (Fig. 5a, c, g, h). In agreement with 4-Di-2-ASP loading assays displayed in Fig. 4, only a subset of hair cells loaded with FM1-43FX in posterior neuromasts with either an A–P or a D–V orientation in *tmc2b* ^*−/−*^ larvae (Fig. 5b, d). Interestingly, we observed that the number of posterior-facing cells with entry of FM1-43FX was significantly higher than anterior-facing cells (Fig. 5g; Supplementary Table 1). A hair cell with posterior-facing hair bundles is defined as one in which the longest stereocilia are more proximal to the head than the shorter protrusions; these hair bundles transduce when deflected anteriorly. From these data, we conclude that in the PLL, hair bundle orientation is in lockstep with molecular components that control mechanotransduction apparatus function, offering the possibility that PCP formation during development modulates proteins that impact transducer action.

### Tmc2b reliance is constant for each neuromast identity

The fluorescent molecule uptake experiments described above were performed on larvae at the same time point, 6-dpf. Therefore, one potential explanation for limited fluorescent molecule uptake is that the neuromast’s Tmc2b reliance is transitioning between levels of dependence, and we observed a time point in that shift. Alternately, the distribution of Tmc2b reliance may be constant. This distinction could be important, as the latter would imply that water flow is being detected by different sets of proteins that directly or indirectly influence transduction at the tips of stereocilia rather than a stepwise process for the developmental regulation of protein expression. To distinguish if this reliance is transient and developmentally related, we repeated the uptake assay on A–P oriented posterior neuromasts of older fish, 14 dpf. The percentage and orientation of hair cells that had partially active mechanotransduction channels were nearly the same as at 6 dpf, indicating that Tmc2b reliance is constant within this time period and not being modulated (Supplementary Fig. 6a; Supplementary Table 1). In addition, examination of D–V-oriented neuromasts in the *tmc2b* ^*−/−*^ mutant’s PLL revealed that hair cells with residual mechanotransduction (dye uptake) are predominantly those that face ventrally, and this dependence is preserved at 6- and 14-dpf (Fig. 5h; Supplementary Fig. 6b; Supplementary Table 1). These results indicate that during early larval stages, Tmc2b reliance is constant with respect to each neuromast position and the particular orientations of hair cells within.

### Tmc2b dependence is non-uniform and asymmetric on head

We next tested whether the asymmetric molecular biases of the trunk and tail neuromasts were recapitulated in the head neuromasts that lie approximately on single planes or sheets on each of the lateral sides of the fish (Fig. 1a, Supplementary Fig. 1) of *tmc2b* ^*−/−*^ mutants. Co-labeling 6-dpf larvae with phalloidin and FM1-43FX demonstrated that hair cells that took up dye within each neuromast were strongly biased for a single direction in all but one neuromast (Fig. 5g, h; Supplementary Table 1). In agreement with 4-Di-2-ASP uptake assays performed on IO4 (Fig. 4d, e), FM1-43FX accumulated in all hair cells irrespective of hair bundle orientation (Fig. 5e, f, h), demonstrating that PCP was not a significant factor in Tmc2b regulation in this particular neuromast. The populations of hair cells that loaded FM1-43FX generally faced posteriorly for A–P-oriented neuromasts of the head (Fig. 5g; Supplementary Table 1). For MI1 and M2, which have different placodal origins and accordingly are innervated by different ganglionic afferents, the uptake pattern was opposite for MI1 (ventral facing) and M2 (dorsal facing) (Fig. 5h; Supplementary Fig. 7; Supplementary Table 1 **)**. This indicates that Tmc2b can be a dominant factor for either direction, dorsal or ventral. These patterns were maintained in 14-dpf zebrafish (Supplementary Fig. 6; Supplementary Table 1). Our studies indicate that each ALL mechanotransduction apparatus is tailored for each neuromast during development, based on the position in the body plan and orientation of the hair cells.

Overall, the molecular differences within the entire complement of neuromasts suggest that not all neuromasts are functionally equivalent. This agrees with a recent finding demonstrating that distinct neuromasts contribute differently to behaviors^31^. The exact parameters impacted by differential Tmc2b expression is an intriguing subject for further investigation.

### Mechanosensory map of Tmc2b reliance

**Fig. 6:**
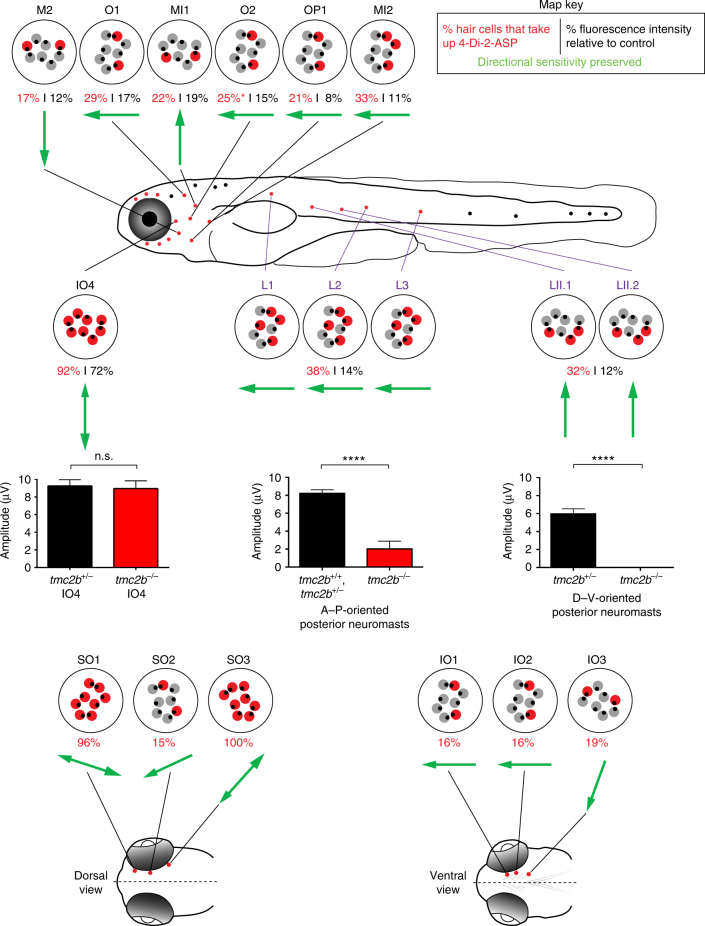
A mechanosensory map of Tmc2b dependence in the anterior and posterior lateral lines. In this graphic of a larval zebrafish *tmc2b* ^*−/−*^ mutant, red neuromasts have been characterized. Individual neuromasts with patterns of FM1-43FX uptake are displayed. Red hair cells take up FM1-43FX; gray hair cells do not. Below each schematized neuromast is the percentage of hair cells that load 4-Di-2-ASP (red text) in the *tmc2b* ^*−/−*^ mutant and the percentage of fluorescence intensity of hair cells of the mutant relative to hair cells of wild-type and heterozygous animals (black). Green arrows represent the directional sensitivity preserved in the mutant based on FM1-43FX uptake. Graphs of mean microphonic potentials from neuromasts directly above each plot are displayed. For IO4, *tmc2b* ^+/*−*^ = 9.2 ± 0.7 μV (*n* = 9), and *tmc2b* ^*−/−*^ = 8.9 ± 0.87 μV (*n* = 6). *P* value = 0.9546. For A–P oriented posterior neuromasts, *tmc2b* ^+/+^, *tmc2b* ^+*/−*^ = 8.3 ± 0.37 μV (*n* = 20), and *tmc2b* ^*−/−*^ = 2.1 ± 0.8 μV (*n* = 15). ****P value < 0.0001. For D–V oriented posterior neuromasts, *tmc2b* ^+*/−*^ = 6 ± 0.49 μV (*n* = 4) and *tmc2b* ^*−/−*^ = 0 ± 0 μV (*n* = 6). ****P value = 0.0048. *P* values were obtained from the Mann–Whitney test. For 4-Di-2-ASP uptake assays of SO and IO neuromasts, *n* ≥ 5. *n* values for other neuromasts are listed in Figs. 3, 4 legends

To visualize the complex anatomical and molecular associations of the lateral line as they relate to Tmc2b, we generated an integrated map of Tmc2b reliance (Fig. 6). Through this *mechanosensory map*, which displays neuromast position, hair cell orientation, and quality of mechanotransduction, a molecular logic for water flow detection begins to emerge. For all observed A–P-oriented neuromasts, partially functional mechanotransduction channels were predominantly from posterior-facing hair cells in *tmc2b* ^*−/−*^ mutants (Fig. 6). The breaks in these neuromasts’ symmetries indicate that detection of water flow from the front of the fish is heavily dependent on Tmc2b. But, encoding water flow coming from the rear relies on a different molecular mechanism that directly or indirectly involves Tmc2b and another factor.

The map (Fig. 6) also demonstrates that Tmc2b dependence varies with specific topological positions of neuromasts. M2 and MI1 had opposite residual sensitivities, indicating that Tmc2b can be a dominant factor for the detection of water flow coming from the top or bottom of the fish (Fig. 6, Supplementary Fig. 7). IO4 is nearly independent of Tmc2b (Fig. 6, Fig. 4d–g, Fig. 5e, f, Supplementary Fig. 2h). Loss of Tmc2b in IO4 does not affect microphonic potentials, which are the result of small cation entry, but entry of the bulky cationic fluorophore is mildly impacted (4-Di-2-ASP^+^ atomic mass = 394 u) (Fig. 6, Fig. 4g, Supplementary Fig. 2h). These findings indicate that IO4 hair cells are minimally dependent on Tmc2b, and this modest reliance is not impacted by hair bundle orientation; however, IO4 may depend on another yet unidentified protein.

Characterization of neuromasts above the eye demonstrated that these neuromasts, whose axes of best sensitivity are tangential to the perimeter of the eye, have variable fluorescent molecule uptake patterns. In the *tmc2b* ^−/−^ mutant, SO1 and SO3 are similar to IO4, with all hair cells able to take up fluorescent molecules similarly. However, SO2 has limited uptake from hair cells that mainly face a single direction. This surprising result indicates that despite being derived from the same placode, not all neuromasts have the same molecular components for mechanotransduction (Fig. 6). Similarly, IO1-4, all derived from a common placode, have variable Tmc2b reliance (Fig. 6). Overall, the map demonstrates that neuromasts employ different molecular mechanisms that are required for mechanotransduction to encode water motion. These mechanisms are based on neuromast topological position and hair bundle orientation.

### Tmc2a and Tmc2b coordinate to permit mechanotransduction

**Fig. 7:**
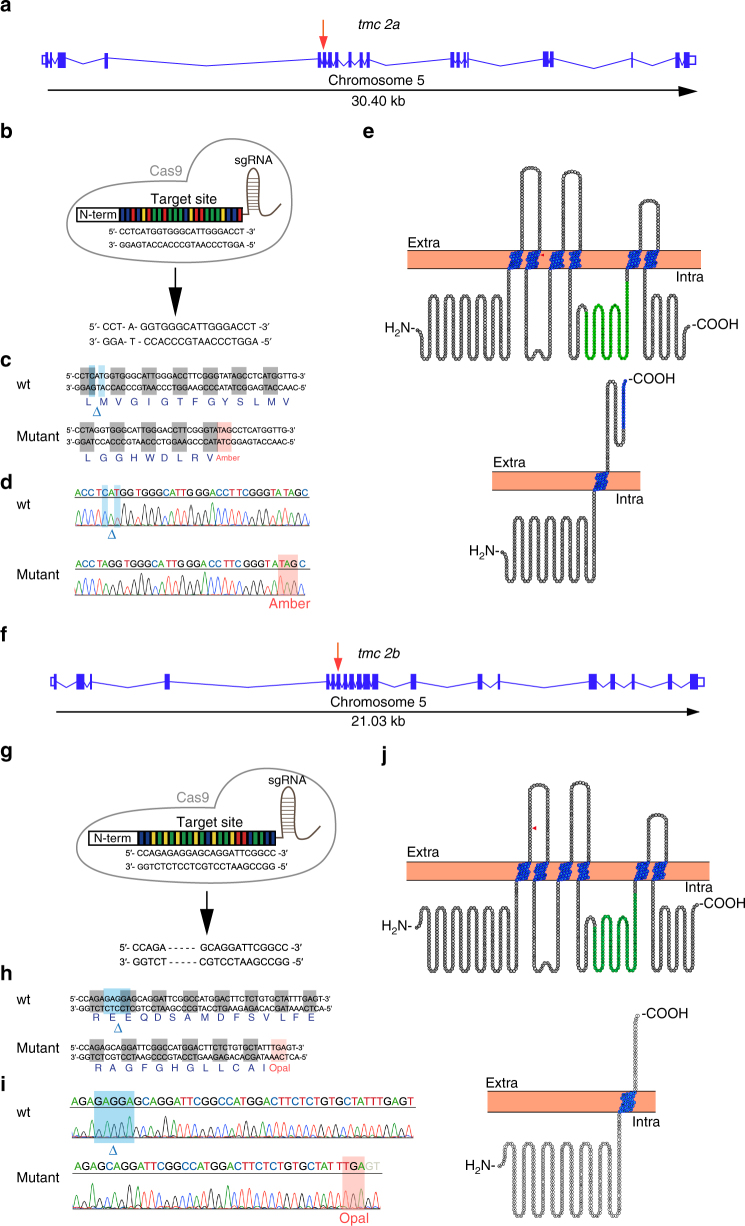
CRISPR-mediated disruption of *tmc2a* and *tmc2b*. Graphical representation of the *tmc2a* **a** and *tmc2b* **f** loci in zebrafish. Putative exons and splice sites are displayed. Red arrows mark the targeted exons. Segments of *tmc2a* exon 6 (**b**) *tmc2b* exon 7 (**g**) were subjected to genome editing. Engineered CRISPR sgRNAs bind target sites to enable DNA cleavage. Mutagenesis deleted 2 nucleotides of *tmc2a* (**b**) and deleted 5 nucleotides of *tmc2b* (**g**), yielding frame-shift mutations. **c**, **h** Amino acid sequences of wild-type and mutant proteins. Sequencing results of mutagenized and control loci, from *tmc2a* (**d**) and *tmc2b* (**i**). Blue highlight and blue delta indicate deleted nucleotides in mutants. Red highlight denotes the stop codons that were generated near the CRISPR-targeting site. **e**, **j** (top) Topographical representations of the Tmc2a (**e**) and Tmc2b (**j**) proteins. Arrowheads indicate points of introduced mutations. Amino acids of transmembrane domains are labeled in blue and the TMC domains are in green. (bottom) Schematics of predicted truncated polypeptides produced in double mutant are displayed

**Fig. 8:**
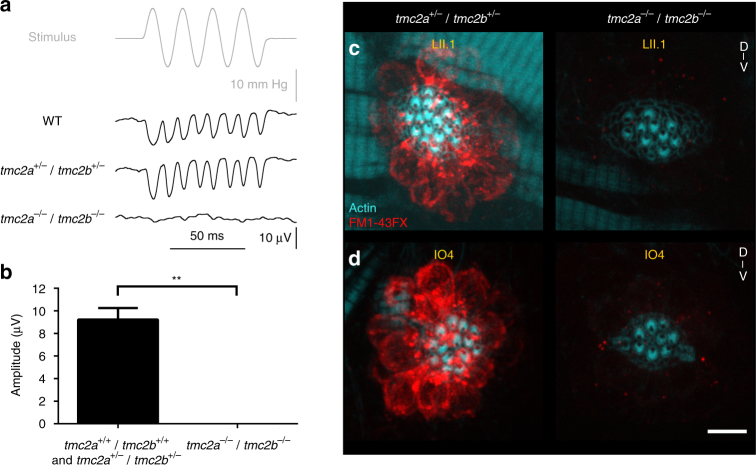
Tmc2a coordinates with Tmc2b to enable mechanotransduction in lateral line hair cells. **a** Stimulus-evoked microphonic potentials measured from posterior neuromasts with A–P-oriented hair cells from 6-dpf zebrafish larvae. The response in *tmc2a* ^*−/−*^ */tmc2b* ^*−/−*^ is absent, bottom trace. **b** Graph of mean microphonic potentials from posterior neuromasts with hair cells with A–P orientations (*n* = 6). **Mann–Whitney test *P* *= *0.0043. **c**, **d** Confocal images of hair cells from LII.1 and IO4 neuromasts of *tmc2a* ^*+/−*^ */tmc2b* ^*+/−*^ or *tmc2a* ^*−/−*^ */tmc2b* ^*−/−*^ animals labeled with FM1-43FX (red) and phalloidin (cyan). No dye was observed in LII.1 or IO4 of *tmc2a* ^*−/−*^ */tmc2b* ^*−/−*^ animals. Scale bar = 6 μm

We next asked what is the identity of the other element that may coordinate with Tmc2b to allow ion entry. To answer this question, we turned to a promising paralogous candidate gene, *tmc2a*, known to be expressed at low levels in the lateral line^15^. Tmc2a and Tmc2b have a 69% amino acid sequence similarity. To determine if *tmc2a* and *tmc2b* genetically interact to enable mechanotransduction, we successfully lesioned both of these genes, which reside on Chromosome 5, separated by ~39.9 centiMorgans. This was accomplished by the simultaneous injection of customized single-guide RNAs (sgRNAs) directed towards exon 6 of *tmc2a* and exon 7 of *tmc2b* along with Cas9 (CRISPR-associated protein 9) mRNA (Fig. 7). Fish with a 2-bp deletion in exon 6 of *tmc2a* and a 5-bp deletion in exon 7 of *tmc2b*, which lead to premature amber (UAG) and opal (UGA) stop codons upstream of regions that encode most transmembrane domains in the respective genes, were characterized. Mechanotransduction quality in double-knockout zebrafish homozygous (*tmc2a* ^*−/−*^ */tmc2b* ^*−/−*^) for the mutations and in controls were assessed using fluorescent molecule uptake and electrophysiological measurements. Stimulus-evoked microphonic potentials were not detectable in neuromasts with A–P orientations in the PLL of *tmc2a* ^*−/−*^ */tmc2b* ^*−/−*^ larvae (Fig. 8a, b) at 6 dpf, yet robust signatures were apparent in controls. This finding was buttressed in an FM1-43FX uptake experiment, where no significant fluorescence was detected in posterior D–V-oriented LII.1 or head D–V-oriented IO4 neuromasts in *tmc2a* ^*−/−*^ */tmc2b* ^*−/−*^ animals (Fig. 8c, d). Enumeration of hair cells in *tmc2a* ^*−/−*^ */tmc2b* ^*−/−*^ mutant larvae revealed a small reduction (~25%) in the numbers of hair cells of posterior neuromast relative to controls (Supplementary Fig. 8). Overall, these findings indicate that the residual activity in the *tmc2b* ^*−/−*^ mutant is dependent on Tmc2a.

**Fig. 9:**
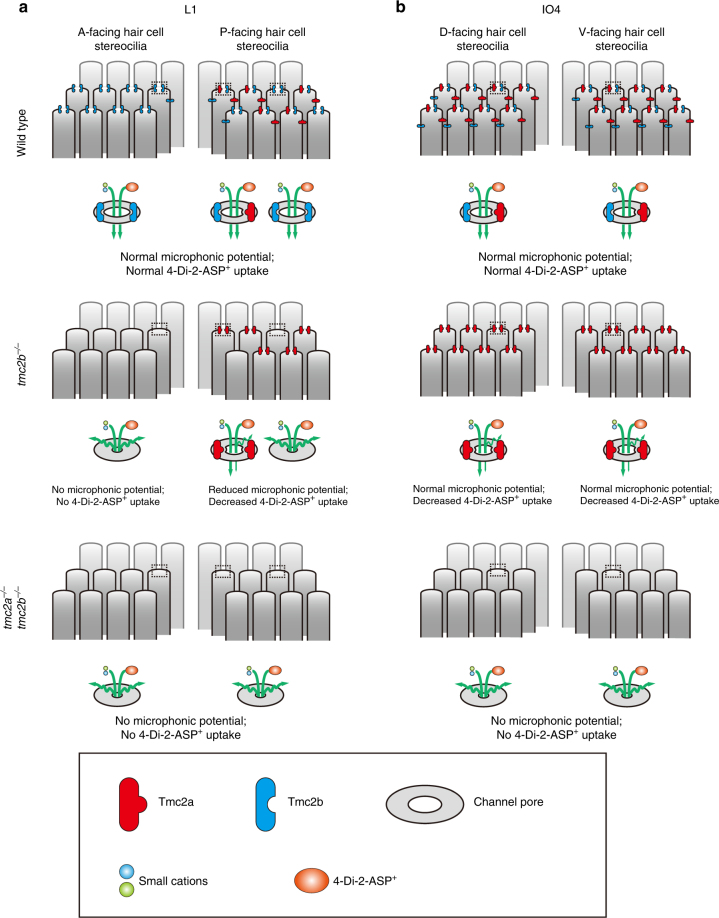
Hypothetical roles of Tmc2b and Tmc2a in neuromast hair cells. **a** In wild-type fish, for one type of L1 neuromast variant, anterior-facing hair bundles depend completely on Tmc2b, but the posterior-facing hair cells depend on Tmc2b and a limited quantity of Tmc2a. In *tmc2b* ^*−/−*^ mutants, hair cells that take up fluorophore predominantly face posteriorly and contribute to microphonic potentials. Asymmetric uptake and microphonic potentials may be due to limited Tmc2a compensation, which permits the decreased passage of fluorophore and the diminished entry of small cations because fewer functional channels assemble. Those that do form have pores with decreased capacity for 4-Di-2-ASP entry. L1 hair cells do not function if they lack both Tmc2b and Tmc2a. **b** In wild-type fish, in this hypothetical model, each mechanotransduction apparatus of IO4 hair cells has  a similar molecular composition irrespective of hair bundle orientation, influenced by high levels of both Tmc2b and Tmc2a. In *tmc2b* ^*−/−*^ knockout fish, Tmc2a compensates for Tmc2b, resulting in a modest change in channel pore quality. IO4 hair cells do not function if they lack both Tmc2b and Tmc2a. Note, these models do not distinguish between Tmc2a and Tmc2b as accessory proteins of the mechanotransduction apparatus or as pore loop-containing channel subunits. They do however explain how mechanotransduction may be impacted by Tmc2a and Tmc2b

Hypothetical models to explain the relationship between neuromast topological position, hair bundle orientation, and Tmc2a and Tmc2b dependencies are displayed in Fig. 9 and Supplementary Fig. 9. These models show how varied Tmc2a and Tmc2b protein expression may give rise to the range of observed phenotypes. Figure 9 models the differences between L1 and IO4. Whereas, Fig. 9a and Supplementary Fig. 9 explain the subtle variations found in L1.

Taken together, our studies suggest that the basis of water motion detection is the coordination of specific mechanotransduction components with discrete neuromast positions and cellular orientations throughout the lateral line. First, we demonstrate that most ALL and PLL neuromasts of the larval zebrafish have maximum sensitivities along two axes, despite having a complicated arrangement of neuromasts on the head (Fig. 1, Supplementary Fig. 1). Second, there are three classes of hair cells with regard to Tmc2b reliance in the lateral line system: absolute, partial, and independent. This suggests that, along with the fact that hair cells with opposite bundle orientations are innervated by different afferents, there is an additional layer of regulation—at the molecular level—in mechanotransduction (Figs. 3, 4). Third, our findings demonstrate that most neuromasts are heavily dependent on Tmc2b, but IO4 is not, suggesting that the developmental factors that result in neuromast placement can govern mechanotransduction channel apparatus function (Fig. 4). Therefore, a developmental plan may predetermine distinct sets of mechanotransduction apparatus components for the neuromast to function properly at its specific topological position. Fourth, hair cells with partial reliance on Tmc2b for mechanotransduction within a single neuromast generally face one direction, indicating that the factors that regulate PCP may also modulate mechanotransduction apparatus composition (Fig. 5). This is functionally evident in the loss of the 2f response in stimulus-evoked microphonic potentials from posterior neuromasts as well as in fluorescent molecule uptake studies. Fifth, interestingly, not all neuromasts that are derived from the same placode demonstrate the same directional dependence on Tmc2b (Fig. 6), suggesting complex modes of Tmc2b regulation even within the same branch of the lateral line.

We speculate that the use of multiple different channel subunits or accessory proteins to change mechanotransduction channel quality may have important consequences for the fish’s ability to detect the direction of water flow. One axis of best sensitivity has hair cells that face anteriorly and posteriorly, but water flowing in either direction may require different responses than a system composed of perfectly symmetrical subunits could provide. The lateral line seems to resolve this conundrum by breaking neuromasts’ morphological mirror symmetries at the molecular level to generate functional asymmetries. Our data suggest that asymmetries are achieved by using at least two sets of components, which contain Tmc2b or both Tmc2a and Tmc2b, to directly or indirectly influence the mechanotransduction channel apparatus, allowing changes in mechanosensory properties for one direction vs. the other. The mechanotransduction parameters affected by differential Tmc2b dependence are an intriguing consideration for the future, as is whether Tmc2b is a member of the mechanotransduction channel itself or if it plays more of an accessory role in the neuromast.

The cochlea is known to have subtle changes in the properties of mechanotransduction channel conductance along the tonotopic gradient, which are considered important for frequency selectivity^32–34^. Mice lacking functional TMC1 largely lose the tonotopic gradient in conductance of outer hair cells^19^. Our findings in the neuromast demonstrate that mechanotransduction channel properties can be modulated by a single mechanotransduction apparatus component, Tmc2b. And, its absence results in three different outcomes, no, partial, and normal transduction, depending on hair cell location and orientation. This suggests that the neuromast and the cochlea may have evolutionarily preserved mechanisms of mechanotransduction conductance modulation—the mixing and matching of mechanotransduction apparatus components. But the neuromast also has another class of hair cell, perhaps an archaic class, with a simpler requirement for mechanotransduction: absolute dependence on Tmc2b. This hair cell class in this respect is similar to *Drosophila* mechanosensitive neurons, which rely on just one TMC protein for mechanotransduction-based larval locomotion^35^ and food texture sensation^36^.

## Methods

### Zebrafish

Wild-type *Tübingen* (*Tü*), *cdh23* ^*aj64a/aj64a*^ mutant^29^, and *Tg(pvalb3b:β-actin-mCherry)* ^37^ zebrafish were used in this study. The CWRU IACUC approved all animal use.

### Molecular biology

PCR was used to amplify *tmc2b* cDNA from zebrafish macular cDNA (Superscript III, Invitrogen) using primers Tmc2b-TA-5′-Cterm (5′-GGGATGGAAATGGATGCAGAATTTGATAGT-3′) and Tmc2b-TA-3′-Cterm (5′-ATCTTCGTGGTGGTGGTCCTCC-3′). The amplicon was ligated into pCR4 TOPO vector (Invitrogen) and digested with *Age*I and *Pme*I before being shuttled, with T4 DNA ligase (Promega), into pMT/pV3b/mGFP vector^38^, which had been digested with *Xma*I and *Pme*I, resulting in pMT/pV3b/tmc2b-mGFP.

### Imaging Tmc2b localization

*Tg(pvalb3b:β-actin-mCherry)* zebrafish injected with DNA plasmid, pMT/pV3b/tmc2b-mGFP, at 150 ng/μl and Tol2 RNA at 25 ng/μl^39^ were anesthetized in 650 mM 3-aminobenzoic acid ethyl ester methanesulfonate (Sigma-Aldrich) in fish water at 4 dpf. Immobilized fish were imaged by confocal microscopy with a 40× objective (Leica SP8; Leica). 488 nm and 594 nm wavelengths were used to excite mGFP and mCherry, respectively.

### Image analyses

Enumeration of the numbers of hair cells that loaded with 4-Di-2-ASP were determined by individual hair cell fluorescent signal on a confocal microscope and compared to the total number of hair cells as determined from bright-field imaging (Fig. 3d, e, Fig. 4f, and Fig. 6).

For the PLL (Fig. 3f), raw images gathered using a microscope (Olympus BX51WI) were subjected to fluorescence measurements using ImageJ. A ROI, a circle 1 μm in diameter, was used to obtain measurements from the brightest cell in each neuromast (*I*_cell_) and an area without cells (*I*_background_) in the same image. Fluorescence intensity of 4-Di-2-ASP-loading (*I*_loading_) for each neuromast was normalized using the formula, *I*_loading_ = *I*_cell_−*I*_background_. To avoid pixel saturation, raw images were taken with three different filter sets that allowed different amounts of light to enter each sample. The relation between incoming light and emitted fluorescence recorded by camera was approximately linear; therefore, *I*_loading_ measured from images taken with a darker filter (6% of light passes), lighter filter (25% of light passes), and no filter (100% of light passes) were corrected by dividing each measurement by a factor of 1, 4.17, or 16.7, respectively, to obtain *I*_corrected_^40,41^. Values of *I*_corrected_ were plotted and subjected to statistical tests.

For the ALL (Fig. 4g), the mean intensities of 4-Di-2-ASP fluorescence were measured from the maximum projection of a *z*-stack image that was collected on a confocal microscope (Leica TCS SP8, Leica) for each neuromast. ROIs were drawn for individual neuromast manually, and the outline of the whole neuromast was defined by the bright-field image taken simultaneously with a fluorescent channel. Intensity values are using a 0–225 range scale set by Leica software without further processing, and no neuromast images with saturated pixels were used in fluorescence quantitation. *I*_whole_ = *I*_neuromast_–*I*_background_.

To quantitate 4-Di-2-ASP fluorescence intensity profiles for multiple individual hair cells within a single neuromast (Supplementary Fig. 4 and Fig. 4c, e), the basolateral region for each hair cell soma was used. The outlines of individual hair cells (ROI) were defined manually by the bright-field image taken simultaneously with the confocal z-stack fluorescent image series (Leica TCS SP8, Leica). The 4-Di-2-ASP fluorescence intensity of each hair cell was the mean pixel value measured within each ROI after subtracting the background pixel intensity value from a 6 × 6 μm region devoid of hair cells (LAS X; Leica).

For Fig. 5g, h and Supplementary Fig. 6, FM1-43FX loading %=(number of A-, P-, D-, or V-facing hair cells that loaded/total number of hair cells with the same orientation)×100.

### Mutation of the *tmc2b* locus with TALENs

To identify a suitable region of exon 4 of the *tmc2b* gene (ENSDARG00000030311) for targeting by transcription activator-like (TAL) effector nucleases (TALENs), the ZiFiT Targeter computational program^42,43^ was used. Specificity of the *tmc2b* TALEN target site was confirmed by the NCBI BLAST tool using the zebrafish genomic DNA database (https://blast.ncbi.nlm.nih.gov/Blast.cgi). TALEN expression vectors were constructed with the fast ligation-based automatable solid-phase high-throughput (FLASH) assembly protocol^25,44,45^. Biotinylated α units, extension units, termination units, and the expression vectors used in this study are listed in order for *tmc2b*-Left-TALEN (TAL373, TAL145-TAL72-TAL203, TAL274, JDS70) and *tmc2b*-Right-TALEN (TAL374, TAL10-TAL32-TAL140, TAL302, JDS71). Designed vectors were confirmed by DNA sequencing using primers oSQT1 (5′-AGTAACAGCGGTAGAGGCAG-3′), oSQT3 (5′-ATTGGGCTACGATGGACTCC-3′), and oJS2980 (5′-TTAATTCAATATATTCATGAGGCAC-3′). Sequence-confirmed expression vectors carrying either *tmc2b*-Left-TALEN or *tmc2b*-Right-TALEN were linearized with *Pme*I and were used as templates for in vitro transcription reactions (Ambion mMessage mMACHINE Ultra T7 kit; Life Technologies Inc.). RNAs were purified by lithium chloride precipitation, and the quality and quantity of each was verified (Agilent 2100 Bioanalyzer, Agilent Technologies; Nanodrop 2000, Thermo Scientific). RNAs that encode each half site of *tmc2b*-TALEN at 150 ng per μl were mixed with phenol red and 1× Danieau’s solution (58 mM NaCl, 0.7 mM KCl, 0.4 mM MgSO_4_, 0.6 mM Ca(NO_3_)_2_, and 5.0 mM HEPES pH 7.6) and then coinjected into zebrafish embryos at the one-cell stage to create gene-specific mutations. Embryos injected with *tmc2b*-TALEN RNAs were collected individually and subjected to mutation detection at the *tmc2b* TALEN target site using high resolution melting analysis (HRMA)^46^ with primers *tmc2b* HRM F (5′-GACGACGAGTCTATGTCTGAAGG-3′) and *tmc2b* HRM R (5′-CAGGTTCACAGCACAAACAGCAAG-3′). The mutations in *tmc2b* carried by founder fish and F1 fish were identified using sequencing primers *tmc2b* SEQ F (5′- CAGCATTCGATGAAATTCTTCATG-3′) and *tmc2b* SEQ R (5′- GAAGGGTAAGGGCAATGCAGTA-3′). *tmc2b* TALEN mutant fish, with a 7-bp deletion (Fig. 2e), used for assays in this work, were genotyped by two sets of primers, *tmc2b* WT SEQ F (5′-CAGCATTCGATGAAATTCTTCATG-3′) and *tmc2b* WT SEQ R (5′- GAGTCTCCTCTTCATGCGCCAG-3′) for the wild-type allele and *tmc2b* mut SEQ F (5′-CTTAGGAACAAACCCTGAAGA-3′) and *tmc2b* mut SEQ R (5′ - CTACCCTGTAAAGGCAATC-3′) for the mutant allele.

### Measurement of neuromast microphonic potentials

Zebrafish larvae were anesthetized using ethyl 3-aminobenzoate methanesulfonic acid (Sigma-Aldrich) dissolved in a standard bath solution (120 mM NaCl, 2 mM KCl, 10 mM HEPES, 2 mM CaCl_2_, 0.7 mM NaH_2_PO_4_ and adjusted to pH 7.3). The larvae were secured in a recording chamber using dental floss tie downs^47^ and placed under the microscope for observation. We visually monitored blood flow and heart rate to assess the viability of each zebrafish larva. Zebrafish were visualized on an upright microscope (BX51WI; Olympus) equipped with a swing nosepiece and 4 × 0.1 and 100 × 1 NA objectives. We recorded microphonic potentials using a PC-505B amplifier (Warner Instruments) and a PCI-6221 digitizer (National Instruments). Images were observed with a Grasshopper3 CMOS camera (Point Grey) and captured with manufacturer provided software. We recorded from neuromasts of larvae ranging from 5 to 6 dpf. Kinocilia tufts were deflected with a fluid jet^23,48^ delivered via a glass pipette with a diameter of ∼7 µm and controlled by HSPC-1 (ALA Scientific Instruments). The fluid jet pipette was placed ∼50 µm away from the neuromast and used to deliver sinusoidal stimuli of 50 Hz, generated by jClamp software (Scisoft, courtesy of Joseph Santos-Sacchi, Yale University, New Haven, CT, USA). The microphonic potentials were recorded at room temperature (~22 °C). We used a borosilicate glass pipette with a resistance of 3–6 MΩ when filled with standard bath solution and placed near the apical edges of the lateral line neuromasts. Placement of fluid jet and recording pipettes were controlled by a set of micromanipulators (MPC-325; Sutter Instrument). Microphonic potentials were recorded with a jClamp in current-clamp mode, amplified by 20 (SIM983, Stanford Research), and low-pass filtered at 200 Hz. All records represent an average of at least 500 trials.

### Measurement of inner ear microphonic potentials

Recording of microphonic potentials from the inner ear in response to vibratory stimuli was conducted on 8–9 dpf larvae. Anesthetized larvae were adhered to the recording chamber by low melting point agarose (IBI Scientific, Peosta, IA). The recording pipette (prepared as described above), loaded with the standard bath solution, was inserted into the otic capsule to reside in close proximity to both maculae. The vibrations were delivered via a glass stylus with a tip diameter of ∼7 µm using a piezo actuator (PA 4/12, Piezosystem Jena) driven by a power amplifier (ENV 800, Piezosystem Jena). The command 200 Hz sinusoidal stimulus was generated by jClamp. Microphonic potentials were recorded as described above and low pass filtered at 1000 Hz.

### Scanning electron microscopy

Zebrafish larvae (5–6 dpf) were fixed in 2.5% glutaraldehyde containing 0.1 M cacodylate buffer (EMS, Hatfield, PA) supplemented with 2 mM CaCl_2_ for 2 h at room temperature. Then, the samples were dehydrated in a graded series of ethanol, critical-point dried with liquid CO_2_ (CPD 030, BAL-TEC AG, Balzers), and sputter-coated with ~10.0 nm palladium (Desk IV, Denton Vacuum). Samples were imaged with a field-emission SEM (Helios Nanolab 650, FEI).

### Real-time polymerase chain reaction

Total RNA from four 6-dpf wild-type or *tmc2b* ^*−/−*^ mutant zebrafish were separately extracted (RNeasy Mini Kit; Qiagen). 400 ng total RNA from each sample was reverse transcribed to produce cDNA (SuperScript III First Strand Synthesis System for RT-PCR Kit; Invitrogen Life Technologies). The relative quantity of each mRNA was obtained with the comparative threshold cycle (*C*_t_) method. PCR amplification was performed with a 20 μl reaction solution (10 ul of 2× RT^2^ SYBR Green ROX Fast Mastermix (Qiagen), 0.4 μl of forward primer (10 μM), 0.4 μl of reverse primer (10 μM), 5.2 μl nuclease-free water, and 4 μl of diluted template cDNA (400 ng/μl)) in a PCR machine (StepOnePlus real-time PCR system; Applied Biosystems). All reactions were performed in quadruplicate. The PCR conditions were as follows: 95 °C for 10 min then forty amplification cycles (95 °C for 15 s, 60 °C for 1 min). As an internal control, primers for beta-actin were used to amplify in parallel reactions. In all cases, a reverse transcriptase negative control was included. Primer sequences: b-actin RT-PCR F2, 5′-GACCCAGACATCAGGGAGTGATGG-3′; b-actin RT-PCR R2, 5′-AGGTGTGATGCCAGATCTTCTCCAGT-3′; Tmc1 RT-PCR F3, 5′-CAGGTCTCAGAAGTTTGCACTG-3′; Tmc1 RT-PCR R3, 5′- GATCACATCGAACAGCATCG-3′; Tmc2a RT-PCR F3, 5′-ATGGCATGAACTTGGTCCTC-3′; and TMC2a RT-PCR R3, 5′-CAGTTTTCCGAGGAATGGAC-3′. Relative amounts of each mRNA were determined by the *C*_t_ strategy, with the mean wild-type control value set at 1.0. *C*_t_ values were obtained for each gene mRNA, with *C*_t_ defined as the threshold cycle of the PCR at which the amplified product was first detected. Δ*C*_t_ is the difference in *C*_t_ values between targets and the β-actin internal control. ΔΔ*C*_t_ represents the difference between ΔC_t_s from wild-type and *tmc2b* ^*−/−*^ zebrafish. The fold difference in a target's expression between wild-type and *tmc2b* ^*−/−*^ mutant zebrafish was calculated as 2^−ΔΔCt^.

### Mutation of *tmc2a* and *tmc2b* with CRISPR/Cas9

Because *tmc2a* (ENSDARG00000033104) and *tmc2b* (ENSDARG00000030311) both reside on chromosome 5, generation of double knockout zebrafish was achieved by co-injection of one-cell stage zebrafish embryos with Cas9 RNA and two sgRNAs that target each of the respective genes. CRISPR target sites were selected using the CHOPCHOP web tool (http://chopchop.cbu.uib.no). sgRNAs were synthesized using T7 RNA polymerase (Ambion MEGAscript)^49^. *tmc2a* was targeted with an sgRNA complementary to exon 6 (5′-CCTCATGGTGGGCATTGGGACCT-3′). *tmc2b* was targeted with an sgRNA complementary to exon 7 (5′-CCAGAGAGGAGCAGGATTCGGCC-3′). Successfully targeted loci were identified by HRMA and DNA sequencing. HRMA primers: tmc2a CRISPR HRMA Forward (5′-CAGGGTTACTGCAAGTACTCAG-3′), tmc2a CRISPR HRMA Reverse (5′-GCAATGAATGAAAGAGGGACTC-3′), tmc2b CRISPR HRMA Forward (5′-TGGGCATCCCTTATGGCTC-3′), and tmc2b CRISPR HRMA Reverse (5′-ATGAGGGGAAACTTACAGCTTG-3′). Sequencing primer pairs for genotyping: tmc2a CRISPR seq Forward (5′-GAGACCGGAAATCTCGTGCC-3′), tmc2a CRISPR seq Reverse (5′-CCTTTTGTCACTGAGGGACAAC-3′), tmc2b CRISPR seq Froward (5′-CCTTGTTCTCTTCGGCTTCATG-3′), and tmc2b CRISPR seq Reverse (5′-TGACAGAGGTAACCGGAACTGC-3′).

### 4-Di-2-ASP uptake assay

At 6 or 14 dpf, zebrafish were bathed with 20 μM 4-Di-2-ASP for 30 s and washed three times at 5 min intervals with rinsing solution (0.612 mM of Ethyl 3-aminobenzoate methanesulfonic acid and 0.01 mg/ml BSA in fish water) prior to imaging. In more detail, for fluorescent molecule uptake, the fish were put into baskets and bathed with labeling solution with manual agitation that resulted in fish movements forward and backward, up and down, and side to side, randomly for 30 s. Data was collected with an upright microscope (BX51WI; Olympus) equipped with a 100× objective with a NA of 1 or a confocal microscope with a 40× objective with a NA of 1.3. On the confocal microscope, a white light laser was used for excitation at 470 nm, and fluorescent signals were collected between wavelengths of 550–600 nm. For comparisons with different gains, laser settings under both low and high conditions were kept the same between genotypes.

### FM1-43FX uptake assays

Zebrafish at 6 or 14 dpf were incubated with 5 μM FM1-43FX (Invitrogen), a fixable analog of FM1-43, for 30 s, then washed three times at 5 min intervals with the rinsing solution before overnight fixation with 4% paraformaldehyde (PFA) at 4 °C. More specifically, for FM1-43FX uptake, the fish were put into baskets and bathed with labeling solution with manual agitation that resulted in fish movements forward and backward, up and down, and side to side, randomly for 30 s. Permeabilization was performed with 3% Triton-X100 (Sigma) overnight at room temperature. Blocking with 5% goat serum was carried out for 6 h at room temperature. Following this, samples were rinsed with phosphate-buffered saline (PBS) four times for 5 min. To visualize hair cells, parvalbumin 3 antiserum at 1:600 dilution was used as a primary antibody and Alexa Fluor 633 goat-anti-rabbit IgG (Invitrogen) at 1:200 dilution was used as the secondary antibody. To identify hair-bundle orientation, Alexa Fluor 633 phalloidin at 1:50 dilution was used to label actin filaments. Processed zebrafish samples were mounted and kept in antifade mounting medium (VECTASHIELD; Vector Laboratories). Images were acquired on a confocal microscope with excitation wavelengths of 488 nm for FM1-43FX and 633 nm for Alexa Fluor 633.

### Statistics and software

All statistical analyses were performed using GraphPad Prism 7. Data are reported as mean ± SEM. Comparisons between groups were tested by Mann–Whitney test, Student’s *t*-Test, Kruskal–Wallis test with Dunn’s multiple comparison test, or ANOVA with Holm-Sidak post hoc testing depending on data distribution. All sample *n* values were greater than 5, unless noted. Topographical representations of proteins were generated with software (TMHMM 2.0; http://www.cbs.dtu.dk/~krogh/TMHMM/).

### Data availability

Data supporting the findings of this study are available upon request from the corresponding author.

## Electronic supplementary material

Supplementary Information
