# Supplementary material for: A molecular basis for water motion detection by the mechanosensory lateral line of zebrafish
Source: Nat Commun. 2017 Dec 21;8:2234. doi: 10.1038/s41467-017-01604-2 (PMC5740173; doi:10.1038/s41467-017-01604-2)
Supplement: Supplementary file 1 — Supplementary Information [file 41467_2017_1604_MOESM1_ESM.pdf]

Supplementary Information

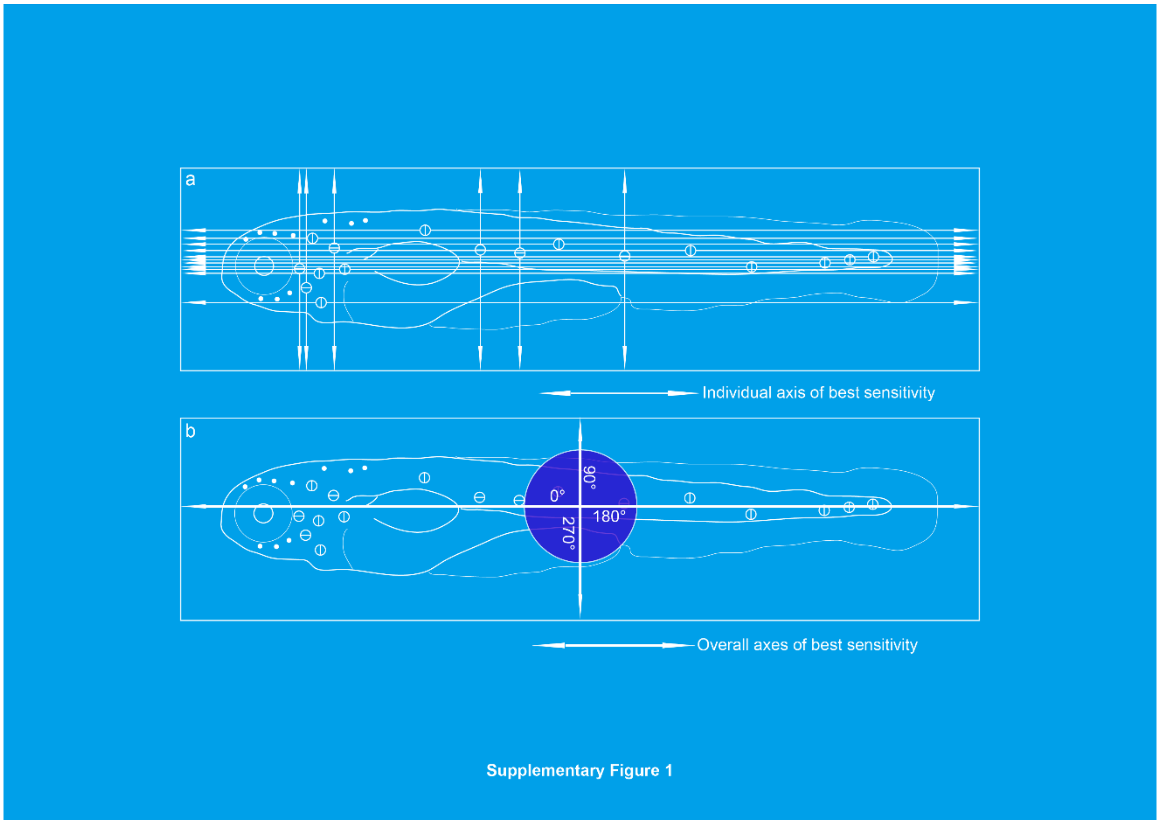

Supplementary Fig. 1. Blueprint of the larval zebrafish's lateral line axes of best sensitivity. (**a**) Schematic of the axes of best sensitivity of neuromasts of the ALL and PLL of larval zebrafish. These neuromasts lay approximately on single planes or sheets on each of the lateral sides of the fish. Posterior neuromasts are slightly offset to show axes of best sensitivity. (**b**) Overall result of perpendicularly set axes of best sensitivity.

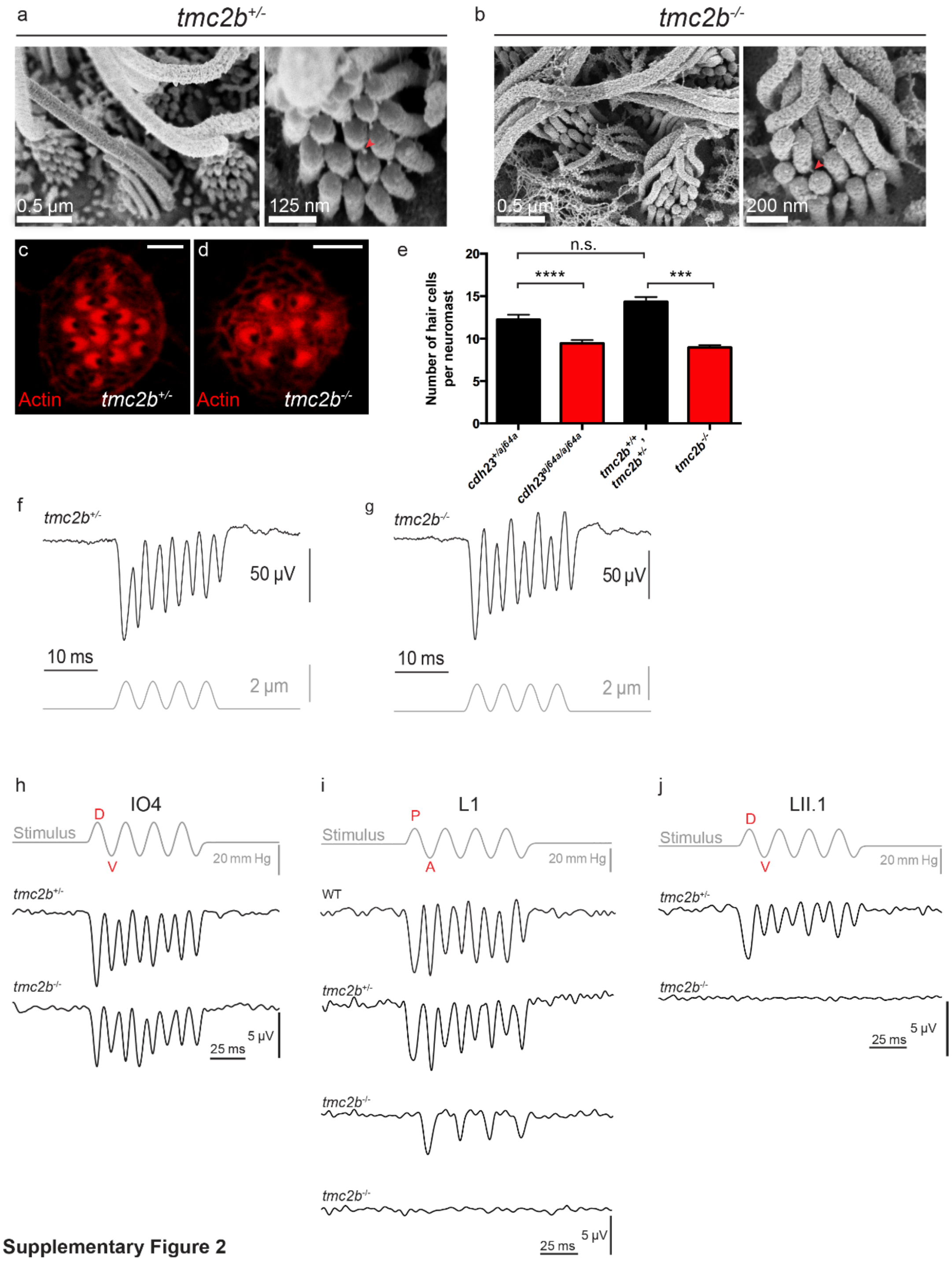

Supplementary Figure 2

Supplementary Fig. 2. Consequences of the *tmc2b* mutation on hair bundle morphology, hair cell presence, and ear and neuromast function. (**a**) Scanning electron micrograph of hair bundles of heterozygous animals with stereocilia (shorter, thinner) and kinocilia (longer, thicker). The stereocilia are linked at their tips by tip link filaments (red arrow head). (**b**) In the *tmc2b*$^{-/-}$ mutant, the hair bundle is morphologically normal at the ultrastructural level. Confocal micrographs of the L2 neuromasts of heterozygous (**c**) and *tmc2b*$^{-/-}$ mutant (**d**) zebrafish labeled with phalloidin (red) at 7 dpf. Planer cell polarity is normal in the mutant. Scale bar = 2.5 µm. (**e**) Mean hair cell numbers ± SEM in posterior neuromasts in *tmc2b*$^{-/-}$, *cdh23*$^{aj64a/aj64a}$, and controls of 6-dpf zebrafish. *cdh23*$^{+/aj64a}$ = 12.3 ± 0.6 (n = 32); *cdh23*$^{aj64a/aj64a}$ = 9.5 ± 0.4 (n = 39); *tmc2b*$^{+/+}$, *tmc2b*$^{+/-}$ = 14.4 ± 0.6 (n = 31); *tmc2b*$^{-/-}$ = 9.0 ± 0.3 (n = 45). **** equal to *P* value < 0.0001 and *** represents *P* = 0.0067, Kruskal-Wallis analysis with a Dunn's multi-comparison test. Representative extracellular recordings of microphonic potentials measured from ears (**f,g**) and neuromasts (**h,i,j**) are displayed (for comparison **i** is the same as in **Fig. 3a**). Ears from controls (67.95 ± 9.52 µV, n = 8) and mutant (69.3 ± 7.86 µV, n = 6) larvae at 8-9 dpf have robust responses at twice the stimulus frequency (*P* = 0.8518, Mann-Whitney test). Microphonic potentials from neuromast IO4 were not impacted by the *tmc2b* mutation. The responses of posterior A-P and D-V oriented neuromasts are most frequently absent (9 of 15 for A-P oriented and 6 of 6 for D-V oriented neuromasts); however, in some cases in A-P oriented, 6 of 15, responses were highly asymmetric, with a weakened amplitude for one direction of stimulus and no response for the other direction. Therefore, the 2f response

became a 1f response. Red letters represent deflection dorsally (D), ventrally (V), anteriorly (A), and posteriorly (P).

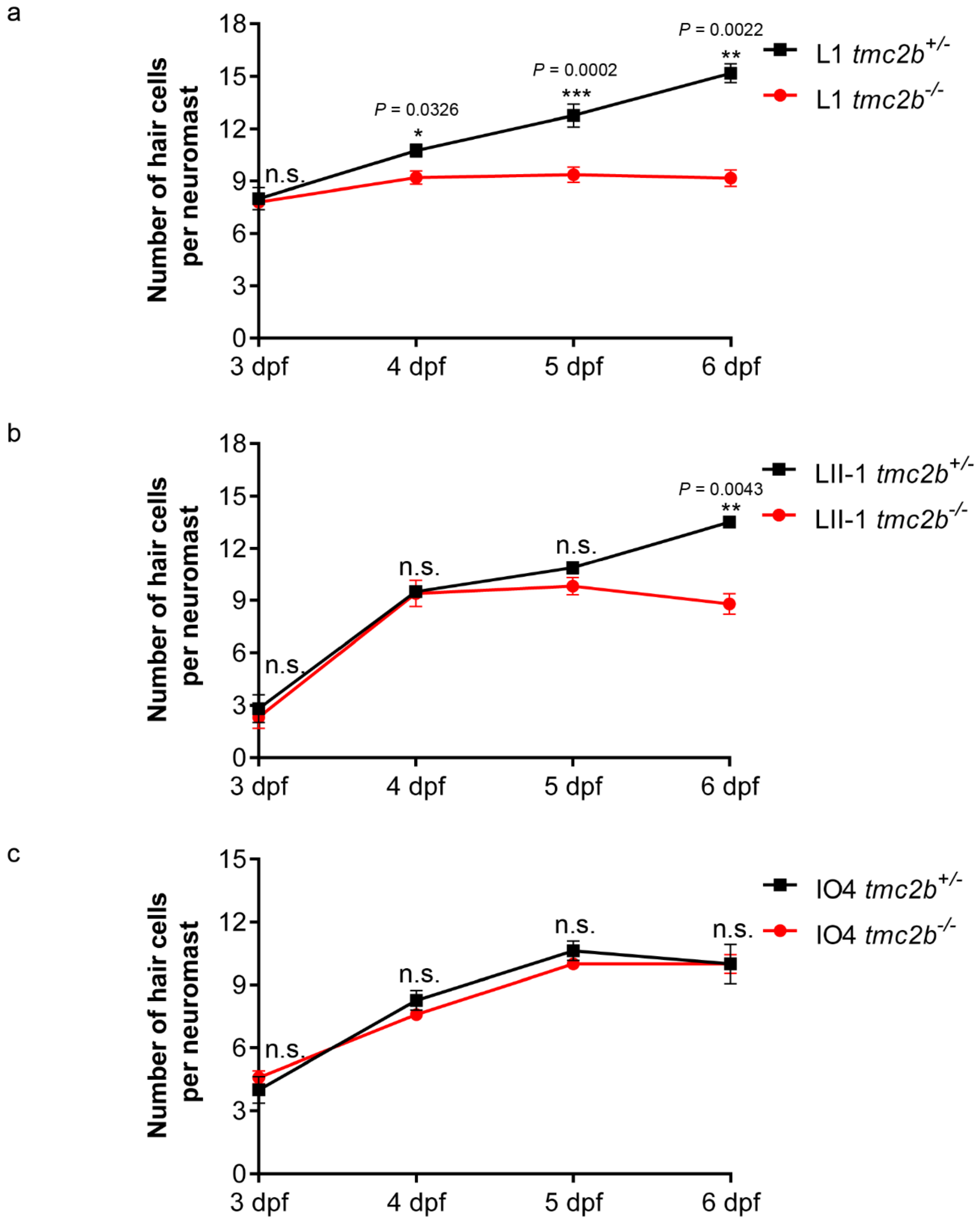

Supplementary Figure 3

Supplementary Fig. 3. Time course of hair cell presence in larval *tmc2b*$^{-/-}$ zebrafish. Hair cell numbers in L1 (**a**), LII.1 (**b**), and IO4 (**c**) neuromasts from 3-6 dpf zebrafish. Mann-Whitney test was used for statistical evaluations. n values are 5, 8, 8, and 6 for *tmc2b*$^{+/-}$ animals at 3 to 6 dpf, respectively. n values are 10, 5, 11, and 6 for *tmc2b*$^{-/-}$ animals at 3 to 6 dpf, respectively.

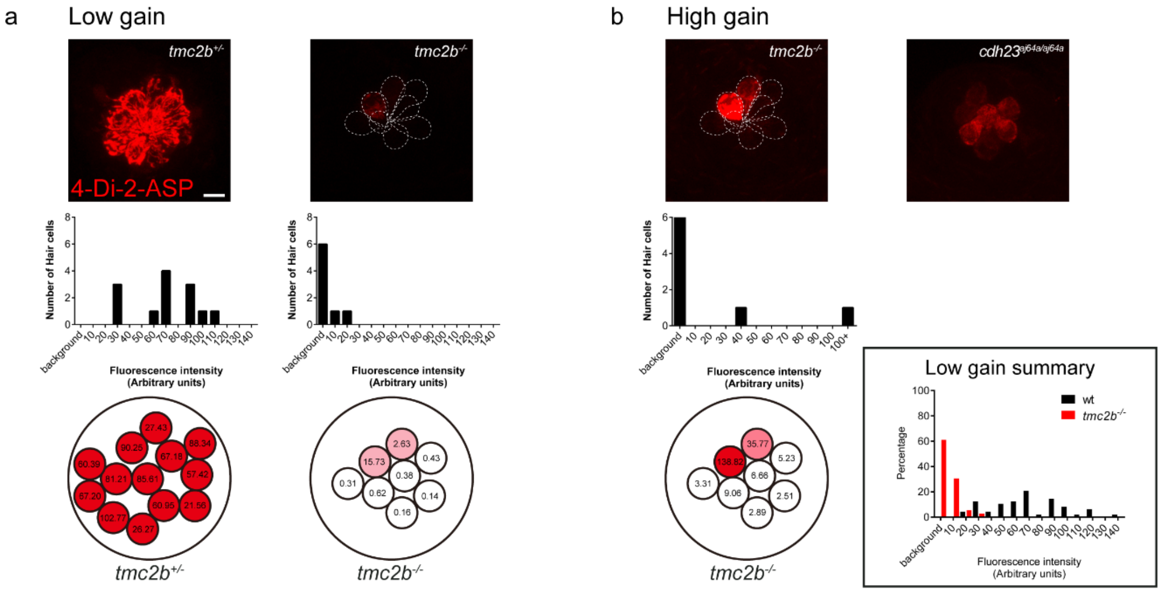

Supplementary Figure 4

Supplementary Fig. 4. Variation of 4-Di-2-ASP uptake in hair cells within a single posterior neuromast of a *tmc2b*$^{-/-}$ mutant. (**a** and **b**) Micrographs of L1 neuromast hair cells exposed to 4-Di-2-ASP (top), histograms of individual hair cell uptake (middle), and quantitative maps of hair cell uptake (bottom) under low gain (**a**) and high gain (**b**). Scale bar = 6 μm. (**a**) Every posterior neuromast hair cell in *tmc2b*$^{+/-}$ larvae take up 4-Di-2-ASP, ranging from 30-110 fluorescence intensity units. In mutants, under low gain, hair cell fluorescence intensity values are negligible (<1 unit) for six cells and very low (1-20 units) for two cells. Background is defined as 0-1 unit. (**b**) Under high gain, 6 cells from a *tmc2b*$^{-/-}$ animal had negligible (<10 units) fluorescence intensities; however, two had increased intensities. In contrast, all hair cells of the *cdh23*$^{aj64a/aj64a}$ posterior neuromasts take

up 4-Di-2-ASP. (**Inset**) Summary, percentages of hair cells at different fluorescence intensity units under low gain (n = 5).

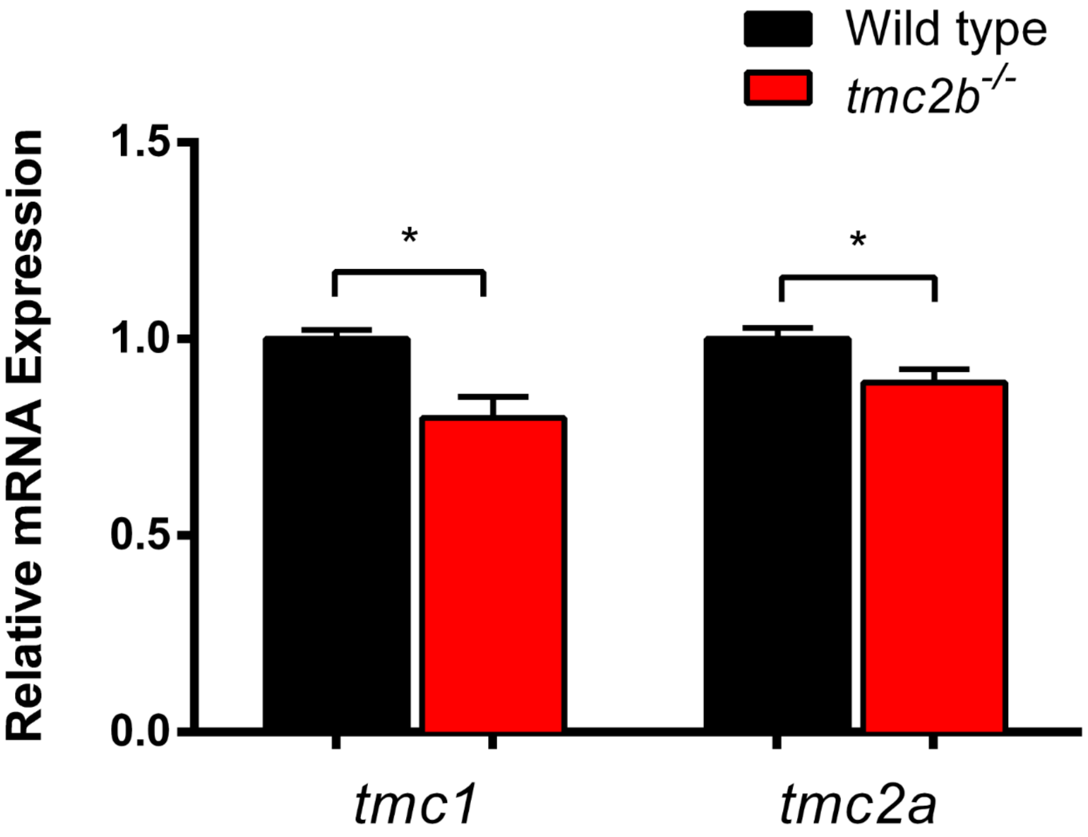

Supplementary Figure 5

Supplementary Fig. 5. Relative *tmc1* and *tmc2a* mRNA levels in the *tmc2b* mutant. Quantitative RT-PCR analyses for *tmc1* and *tmc2a* mRNAs from 6-dpf larvae. * P = 0.0139 and 0.0423 for *tmc1* and *tmc2a*, respectively (n = 4). Unpaired Student's *t*-test used.

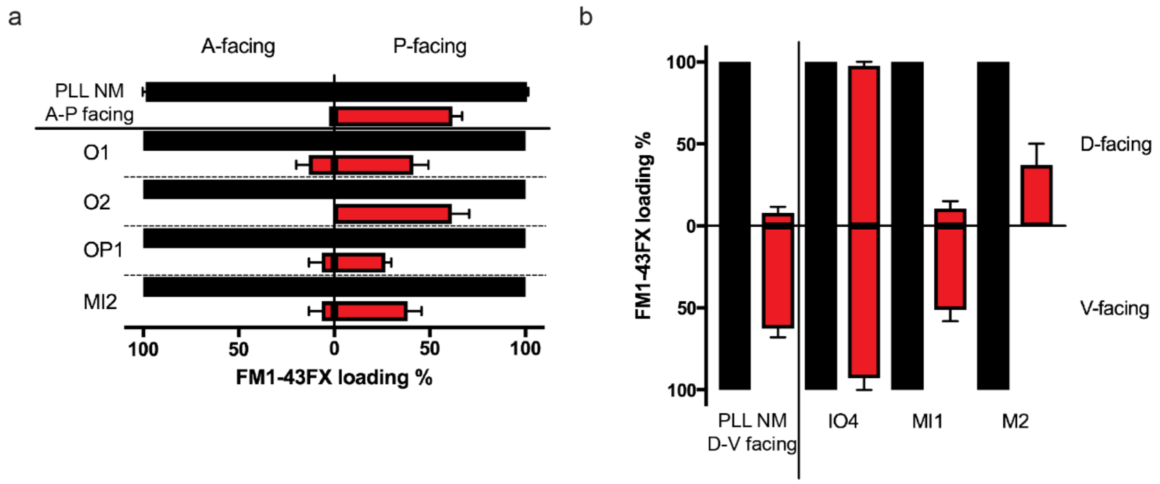

Supplementary Figure 6

Supplementary Fig. 6. Roles of hair bundle polarity and neuromast position on Tmc2b-dependent mechanotransduction channel function in 14-dpf zebrafish. (a and b) Graphs from FM1-43FX-uptake and phalloidin-labeling assays performed on 14-dpf zebrafish. Red bars represent *tmc2b*$^{-/-}$, and black bars signify *tmc2b*$^{+/+}$ and *tmc2b*$^{+/-}$. (**a**) In A-P oriented posterior neuromasts in *tmc2b*$^{-/-}$ mutants, most of the FM1-43FX uptake is by hair bundles that face posteriorly (P-facing). *tmc2b*$^{-/-}$ (A-facing) = 2.6 ± 1.92 % (n = 19), *tmc2b*$^{-/-}$ (P-facing) = 61.8 ± 5.14 % (n = 19), $P < 0.0001$. In *tmc2b*$^{+/+}$ and *tmc2b*$^{+/-}$ fish, each neuromast has similar loading percentages for hair cells of opposing directions. For O1, O2, OP1, and MI2 neuromasts, P-facing hair cells also preferentially uptake fluorophore. (**b**) Hair cells with ventral (V)-facing hair bundles dominate the population of hair cells that pass the FM1-43FX in D-V oriented posterior neuromasts in *tmc2b*$^{-/-}$ mutants. *tmc2b*$^{-/-}$ dorsal (D)-facing = 7.8 ± 3.86 % (n = 15), *tmc2b*$^{-/-}$ V-facing = 62.7 ± 5.36 % (n = 15), $P < 0.0001$. In IO4 neuromasts of *tmc2b*$^{-/-}$, every hair cell takes up

FM1-43FX, unrelated to the direction that the hair bundles face. In contrast, in MI1, it is more probable to have ventrally facing hair bundles that have channels that pass FM1-43FX in *tmc2b*$^{-/-}$ mutants, but in M2 neuromasts dorsally facing hair bundles are more likely to take up the fluorophore. *P* values shown above are by One-way ANOVA with Holm-Sidak's *post-hoc* testing. Equal FM1-43FX uptake for either orientation of hair cells was observed in neuromasts of control animals.

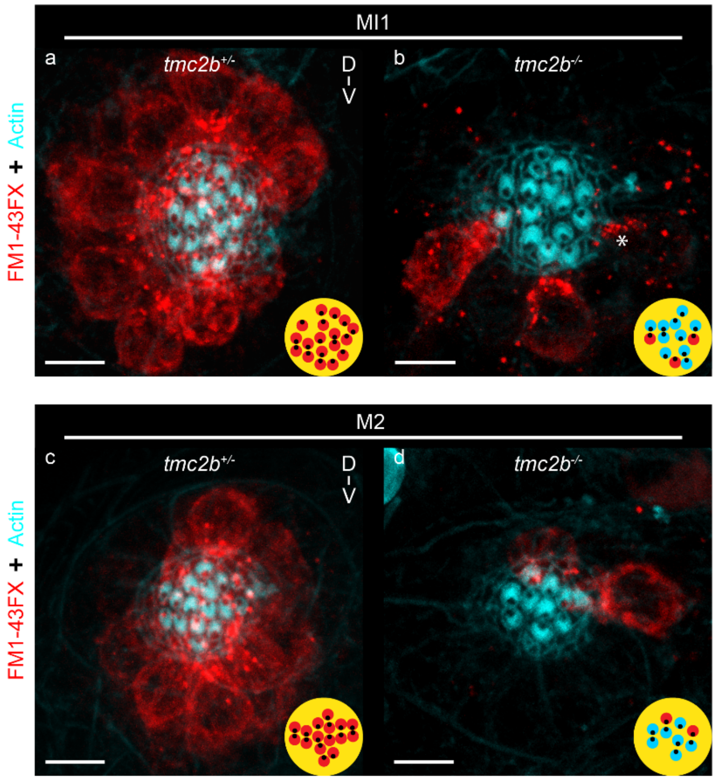

Supplementary Figure 7

Supplementary Fig. 7. Roles of hair bundle polarity and neuromast position in the dependence of mechanotransduction channel function on Tmc2b for neuromasts MI1 and M2. Images of hair cells from neuromasts MI1 (**a,b**) and M2 (**c,d**) of *tmc2b*$^{+/-}$ and *tmc2b*$^{-/-}$ 6-dpf larvae. FM1-43FX (red) presence reveals functional channels, and phalloidin (cyan) shows hair bundle polarity. Qualitative maps (yellow) of micrographs indicate that MI1 hair cells with ventrally facing hair

bundles and M2 hair cells with dorsally facing hair bundles preferentially function (red) in *tmc2b*$^{-/-}$ mutants. Scale bar = 5 μm.

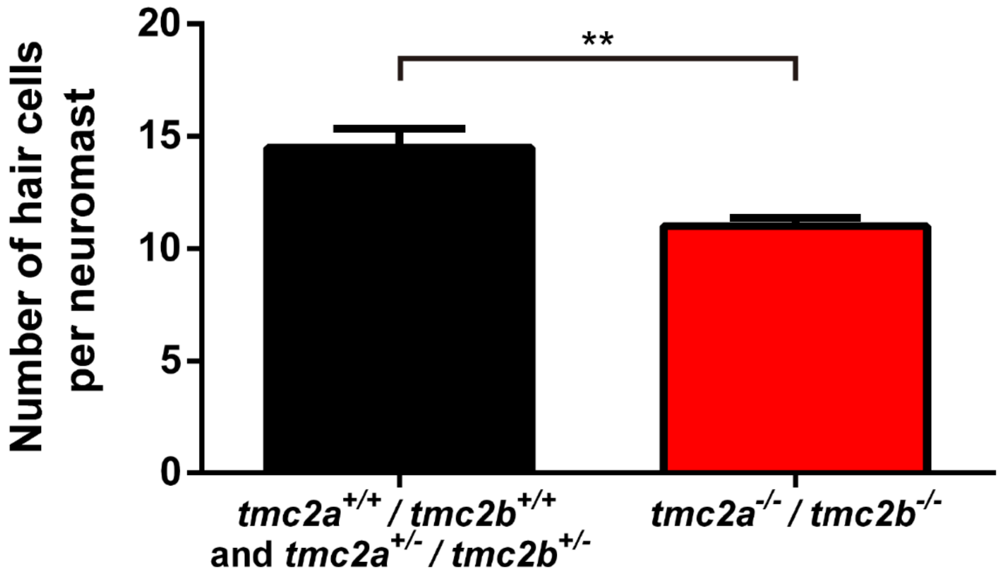

Supplementary Figure 8

Supplementary Fig. 8. Quantitation of hair cell numbers in *tmc2a*$^{-/-}$/*tmc2b*$^{-/-}$ mutant larvae. Using DIC imaging, the numbers of hair cells were enumerated from A-P oriented posterior neuromasts of control and *tmc2a*$^{-/-}$/*tmc2b*$^{-/-}$ mutant larvae at 6-dpf. ** $P = 0.0014$, unpaired Student's *t*-test, n = 8.

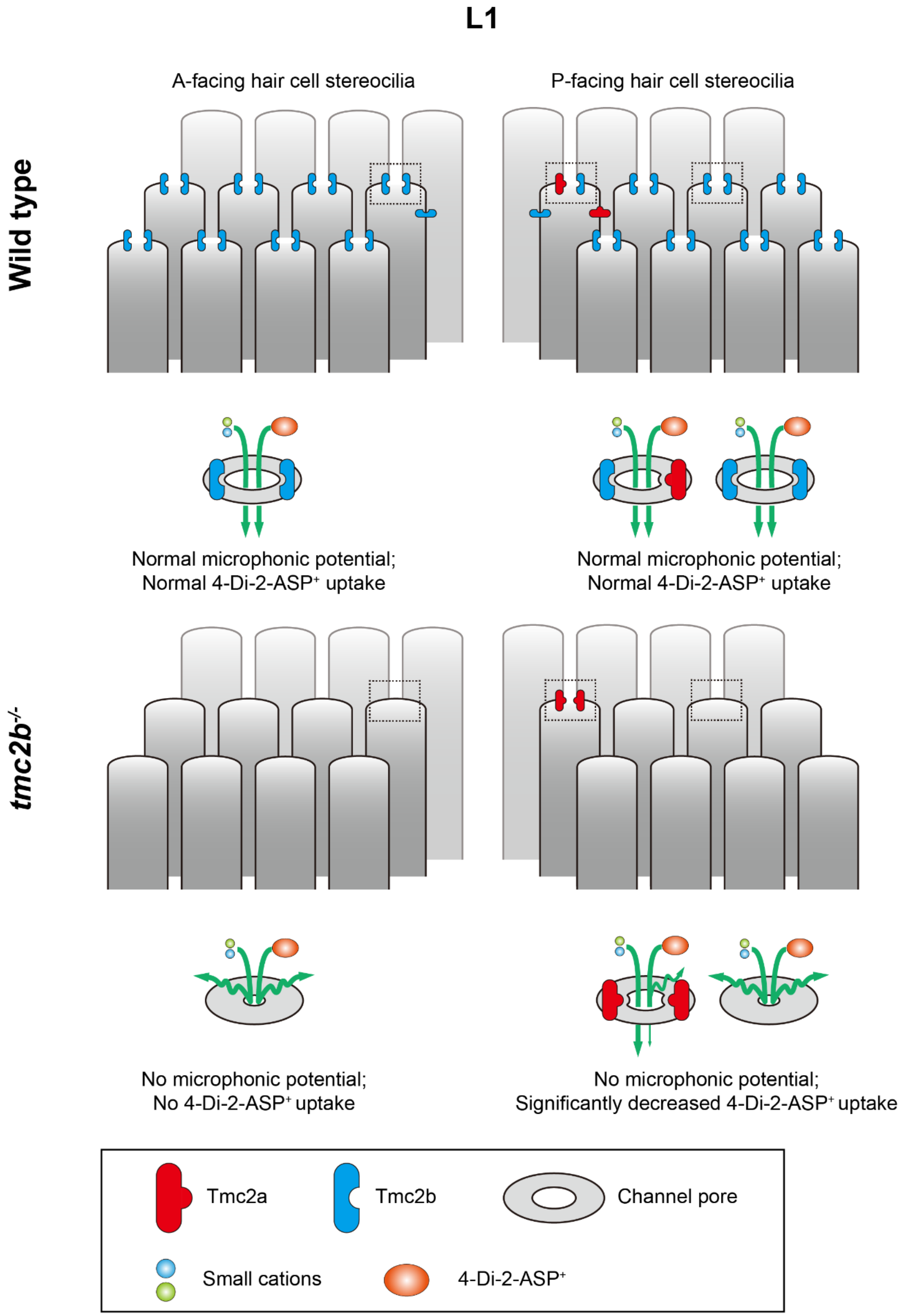

Supplementary Figure 9

Supplementary Fig. 9. A hypothetical molecular model of the roles of Tmc2a and Tmc2b in an A-P oriented posterior neuromast variant. In this model of a L1 variant, the mechanotransduction channels are more heavily reliant on Tmc2b than the model in **Fig. 9**. This model is based on our microphonic potential experiment results (**Fig. 3a bottom trace**). In the L1 neuromast, anterior-facing hair bundles still depend absolutely on Tmc2b. However, posterior-facing hair cells are mostly dependent on Tmc2b with minuscule levels of Tmc2a relative to IO4 and the other type of L1 (**Fig. 9**). In the *tmc2b*$^{-/-}$ mutant, Tmc2a in posteriorly-facing hair cells allows the passage of fluorophore and small cations, but at low quantities, such that microphonic potentials are undetectable.

Supplementary Table 1

Lateral Line structure	Genotype	Percentage of FM1-43FX loading			
		6 dpf		14 dpf	
PLL A-facing hair cells	*tmc2b* $^{+/+}$, *tmc2b* $^{+/-}$	98.4 ± 1.09, n = 16	n.s.	98.7 ± 1.67, n = 15	n.s.
PLL P-facing hair cells	*tmc2b* $^{+/+}$, *tmc2b* $^{+/-}$	98.3 ± 1.15, n = 16		100.7 ± 0.74, n = 15	
PLL A-facing hair cells	*tmc2b* $^{-/-}$	8.1 ± 3.98, n = 21	$P < 0.0001$	2.6 ± 1.92, n = 19	$P < 0.0001$
PLL P-facing hair cells	*tmc2b* $^{-/-}$	64.7 ± 4.42, n = 21		61.8 ± 5.14, n = 19	
O1 A-facing hair cells	*tmc2b* $^{+/+}$, *tmc2b* $^{+/-}$	97.8 ± 2.22, n = 5	n.s.	100 ± 0, n = 5	n.s.
O1 P-facing hair cells	*tmc2b* $^{+/+}$, *tmc2b* $^{+/-}$	100 ± 0, n = 5		100 ± 0, n = 5	
O1 A-facing hair cells	*tmc2b* $^{-/-}$	0 ± 0, n = 4	$P < 0.0001$	13.3 ± 6.67, n = 3	$P = 0.0199$
O1 P-facing hair cells	*tmc2b* $^{-/-}$	44.2 ± 7.9, n = 4		41.3 ± 7.94, n = 3	
O2 A-facing hair cells	*tmc2b* $^{+/+}$, *tmc2b* $^{+/-}$	100 ± 0, n = 5	n.s.	100 ± 0, n = 5	n.s.
O2 P-facing hair cells	*tmc2b* $^{+/+}$, *tmc2b* $^{+/-}$	100 ± 0, n = 5		100 ± 0, n = 5	
O2 A-facing hair cells	*tmc2b* $^{-/-}$	3.3 ± 3.33, n = 5	$P = 0.0011$	0 ± 0, n = 5	$P < 0.0001$
O2 P-facing hair cells	*tmc2b* $^{-/-}$	35.4 ± 7.72, n = 5		61.5 ± 9.1, n = 5	
OP1 A-facing hair cells	*tmc2b* $^{+/+}$, *tmc2b* $^{+/-}$	98 ± 2, n = 5	n.s.	100 ± 0, n = 5	n.s.
OP1 P-facing hair cells	*tmc2b* $^{+/+}$, *tmc2b* $^{+/-}$	100 ± 0, n = 5		100 ± 0, n = 5	
OP1 A-facing hair cells	*tmc2b* $^{-/-}$	3.0 ± 3.03, n = 11	$P = 0.0003$	6.7 ± 6.67, n = 5	$P = 0.0345$
OP1 P-facing hair cells	*tmc2b* $^{-/-}$	26.8 ± 5.78, n = 11		26.7 ± 3.12, n = 5	
MI2 A-facing hair cells	*tmc2b* $^{+/+}$, *tmc2b* $^{+/-}$	100 ± 0, n = 5	n.s.	100 ± 0, n = 4	n.s.
MI2 P-facing hair cells	*tmc2b* $^{+/+}$, *tmc2b* $^{+/-}$	100 ± 0, n = 5		100 ± 0, n = 4	
MI2 A-facing hair cells	*tmc2b* $^{-/-}$	6.7 ± 6.67, n = 5	$P < 0.0001$	6.7 ± 6.67, n = 3	$P = 0.0065$
MI2 P-facing hair cells	*tmc2b* $^{-/-}$	51 ± 9, n = 5		38.3 ± 7.27, n = 3	
PLL D-facing hair cells	*tmc2b* $^{+/+}$, *tmc2b* $^{+/-}$	99.8 ± 2.98, n = 15	n.s.	100 ± 0, n = 17	n.s.
PLL V-facing hair cells	*tmc2b* $^{+/+}$, *tmc2b* $^{+/-}$	101.3 ± 2.48, n = 15		100 ± 0, n = 17	
PLL D-facing hair cells	*tmc2b* $^{-/-}$	3.5 ± 1.91, n = 16	$P < 0.0001$	7.8 ± 3.86, n = 15	$P < 0.0001$
PLL V-facing hair cells	*tmc2b* $^{-/-}$	61.6 ± 5.08, n = 16		62.7 ± 5.36, n = 15	
IO4 D-facing hair cells	*tmc2b* $^{+/+}$, *tmc2b* $^{+/-}$	98 ± 5.56, n = 5	n.s.	100 ± 0, n = 5	n.s.
IO4 V-facing hair cells	*tmc2b* $^{+/+}$, *tmc2b* $^{+/-}$	101.1 ± 5.47, n = 5		100 ± 0, n = 5	
IO4 D-facing hair cells	*tmc2b* $^{-/-}$	83 ± 11.11, n = 5	n.s.	97.6 ± 2.38, n = 7	n.s.
IO4 V-facing hair cells	*tmc2b* $^{-/-}$	82.6 ± 11.08, n = 5		92.9 ± 7.14, n = 7	
MI1 D-facing hair cells	*tmc2b* $^{+/+}$, *tmc2b* $^{+/-}$	100 ± 0, n = 4	n.s.	100 ± 0, n = 5	n.s.
MI1 V-facing hair cells	*tmc2b* $^{+/+}$, *tmc2b* $^{+/-}$	100 ± 0, n = 4		100 ± 0, n = 5	
MI1 D-facing hair cells	*tmc2b* $^{-/-}$	7.9 ± 3.57, n = 6	$P = 0.0022$	10.7 ± 4.4, n = 5	$P < 0.0001$
MI1 V-facing hair cells	*tmc2b* $^{-/-}$	36.1 ± 3.35, n = 6		51.3 ± 6.8, n = 5	
M2 D-facing hair cells	*tmc2b* $^{+/+}$, *tmc2b* $^{+/-}$	100 ± 0, n = 5	n.s.	100 ± 0, n = 5	n.s.
M2 V-facing hair cells	*tmc2b* $^{+/+}$, *tmc2b* $^{+/-}$	98.2 ± 1.82, n = 5		100 ± 0, n = 5	
M2 D-facing hair cells	*tmc2b* $^{-/-}$	44.9 ± 8.3, n = 5	$P < 0.0001$	37.1 ± 13.04, n = 5	$P < 0.0001$
M2 V-facing hair cells	*tmc2b* $^{-/-}$	2.9 ± 2.86, n = 5		0 ± 0, n = 5	

Supplementary Table 1. Side-by-side comparisons of FM1-43FX uptake in neuromasts from 6- and 14-dpf zebrafish. Hair cells with opposing orientations in each neuromast were compared using one-way ANOVA with Holm-Sidak multiple comparisons.
